# Monitoring Water in Vegetation: Mind the Gap Between Satellite Remote Sensing and Field Scale Approaches

**DOI:** 10.1007/s40725-026-00290-y

**Published:** 2026-11-14

**Authors:** David Chaparro, Thomas Jagdhuber, Marta Yebra, Christoph Rüdiger, François Jonard, Andrew F. Feldman, Alexandra G. Konings, Oliver Binks

**Affiliations:** 1 Centre for Ecological Research and Forestry Applications (CREAF) - Fundació Institució dels Centres de Recerca de Catalunya (CERCA), Cerdanyola del Vallès, Barcelona, 08193 Spain; 2 German Aerospace Center, Microwaves and Radar Institute, Münchener Strasse 20, Wessling, 82234 Germany; 3 Institute of Geography, University of Augsburg, Alter Postweg 118, Augsburg, 86159 Germany; 4 Fenner School of Environment and Society, The Australian National University, ACT, Canberra, Australia; 5 School of Engineering, The Australian National University, ACT, Canberra, Australia; 6 European Centre for Medium-Range Weather Forecasts, Bonn, Germany; 7 Earth Observation and Ecosystem Modelling Laboratory, SPHERES Research Unit, Université de Liège (ULiege), Allée du Six Août 19, Liège, 4000 Belgium; 8 NASA Goddard Space Flight Center, Greenbelt, MD USA; 9 Earth System Science Interdisciplinary Center, University of Maryland, College Park, MD USA; 10 Department of Earth System Science, Stanford University, Stanford, CA USA; 11 Research School of Biology, the Australian National University, Canberra, ACT 2601 Australia

**Keywords:** Vegetation water content, Vegetation water potential, Live fuel moisture content, Passive microwave remote sensing, Ecosystem hydraulics, Vegetation optical depth

## Abstract

**Purpose of The Review:**

Understanding vegetation water dynamics is crucial for monitoring ecosystem health, improving fire risk assessment, and modelling land–atmosphere feedbacks. This review examines how satellite remote sensing can help monitor the vegetation water status (with information about water content and water potential) and identifies the major gaps that currently hinder reconciling satellite data with field-based measurements. It aims to establish bridges across spatial scales to enhance the current monitoring framework for vegetation water status from regional to global levels. The paper mostly focuses on passive microwave remote sensing, which is appropriate for sensing water across different vegetation layers at regional to global scales, and at which the amount of research work and data products are limited.

**Recent Findings:**

Recent research has established and expanded global *in situ* and remote sensing datasets of live fuel moisture content (LFMC) and water potential (Ψ). Also, new passive microwave satellite products offer improved sensitivity to canopy water dynamics at coarse scales. However, efforts to reconcile remote sensing and field data are hampered by point-to-pixel scale mismatches and by a limited understanding of how satellite data relates to ecophysiological processes. Emerging concepts such as ecosystem pressure–volume (EPV) curves (with the potential of footprint-scale representativeness) and new satellite VOD-based approaches provide promising pathways to infer water content and water potential from space. Developments in multi-frequency microwave remote sensing further open new opportunities to monitor vertical gradients of water status in plants and to study the sensitivity of microwaves to water across plant tissues.

**Summary:**

Bridging remote sensing and point-scale research in ecohydrology requires harmonized datasets for validation and comparison of products (here we focus on LFMC products) and new frameworks to monitor water content and water potential. In this paper, we review research progress towards these goals. This will enable large-scale monitoring of vegetation water dynamics, advancing our understanding of plant responses to drought for entire ecosystems and improving spatially-resolved predictions of biosphere–atmosphere interactions.

**Supplementary Information:**

The online version contains supplementary material available at https://doi.org/10.1007/s40725-026-00290-y.

## Introduction

Climate change is causing a warmer and drier atmosphere and more frequent and intense droughts, leading to higher atmospheric water demand and in some cases drier soils compared to prior decades [1–3]. These changes constrain the vegetation’s ability to access water resources, which in turn reduces the capacity of vegetation to assimilate carbon and leads to drought-induced tree mortality [4–7]. At the same time, drier vegetation and soils increase fuel flammability, contributing to more frequent, intense, and larger forest fires [8–10]. Water scarcity also affects the central role of vegetation in providing ecosystem services, including climate and weather regulation through land-atmosphere feedbacks. This influences the global carbon, water and energy cycles [11–16]. Hence, there is considerable interest in monitoring water in vegetation and assessing global vegetation health and function.

Monitoring water status and dynamics in vegetation is important for several applications. For instance, the live fuel moisture content (LFMC), which is the percentage of mass of water relative to the plant’s dry mass, is a key parameter for fire risk assessment since it is used to model fuel flammability and fire behavior [17–21]. Plant water content (θ) and water potential (Ψ, [MPa]) are key indicators of plant water status. Water potential, a thermodynamic property describing the free energy of water and thus its tendency to flow, strongly influences plant function and physiological stress, providing essential information about forest vulnerability to drought. Increasing water stress is generally related to lower water volume and more negative water potential. Water potential is linked to the plant water content through pressure-volume relationships. Enhanced monitoring of vegetation water status can improve our representation of the soil-plant-atmosphere water flux, which has a significant effect on land-atmosphere feedbacks and hydrological cycling. This can ultimately result in improved drought monitoring and forecasting, understanding of plant hydraulics, and climate and meteorological models [11, 22].

Vegetation water status is described by several related yet distinct variables. Some quantify the amount of water stored in leaves, stems, or canopies (e.g., θ and LFMC), whereas others describe the energy state of that water and its tendency to move through the soil–plant–atmosphere continuum (Ψ). Table 1 summarizes these variables and highlights those most relevant to remote sensing, ecophysiology, hydrology, fire risk, and drought assessment. Three main methods exist for estimating these variables: *in situ* samples (e.g. [19]), remote sensing [17, 18], and process-based models [20, 21]. In this work, we review the first two and how they are related across scales. *In situ* data typically have limited spatial extent, are temporally sparse, and require substantial collection effort because the measurements are destructive and/or expensive. For instance, extensive LFMC sampling networks have only been deployed at a regional scale in a few regions [38–40]. At a global scale, datasets of *in situ* LFMC [19, 41] and water potential [45] have only been established recently, with most measurements available in Europe and the USA [19, 45]. Because these datasets rely on direct measurements of plants, they can provide benchmarks for the calibration of models [47] and the validation of satellite retrievals [35, 36] (Table 1).

**Table 1:** Variables quantifying water in vegetation, classified in water content and water potential groups. The main variables addressed in this study are indicated in italics. The other variables can be derived from the main ones or are conceptually related: EWT, CWS and RWC are linked to *VWS* and *θ*^1^. The m_g_ is convertible to/from *LFMC* [18]. Right-hand columns cite some examples of remote sensing products and *in situ* datasets/measurements of each variable

	Variable	SI units	Description	Remote sensing	*In situ*
Water content	*Stem water content (θ*_stem_ *)*	m^3^/m^3^	$\:\:\frac{Volume\:of\:water}{Stem\:volume}$	N/A^2^	[23]
	*Leaf water content (θ*_leaf_ *)*	kg/m^2^	$\:\:\frac{Fresh\:m.\:\:-\:Dry\:m.}{Leaf\:area}$		
	*Vegetation Water Storage (VWS)*	kg/m^2^	$\:\:\frac{Fresh\:m.\:\:-\:Dry\:m.}{ground\:area}$	[24–27]	
	Equivalent water thickness (EWT)	mm	EWT (water column) is a synonim of VWS since water density is 1 g/cm	[28]	[29]
	Canopy water storage (CWS)	kg/m^2^	${\theta}_{leaf}\cdot Leaf\:Area\:Index$	[30, 31]	
	Relative water content (RWC)	%	In plant physiology (RWC_phys_)^1^: $\frac{Fresh\:m.-Dry\:m.}{Turgid\:m.-Dry\:m.}\cdot100$ In remote sensing (RWC_RS_)^3^: $\frac{VOD-{m}{i}{n}\left(VOD\right)}{{m}{a}{x}\left(VOD\right)-{m}{i}{n}\left(VOD\right)}\cdot100$	[32, 33]	[34]
	*Live fuel moisture content (LFMC)*	%	$\frac{{Fresh\:m}_{leaves}-{Dry\:m}_{leaves}}{{Dry\:m}_{leaves}}\cdot100$	[17, 35–37], Table 2	[19, 38–41]^4^
	Gravimetric moisture (m_g_)	kg/kg	$\:\frac{{Fresh\:m}_{leaves}-\:{Dry\:m}_{leaves}}{{Fresh\:m}_{leaves}}$	[35, 42]	
Water potential	*Leaf water potential (Ψ*_1_ *)*	MPa (MJ/m^3^)	Potential energy of water in the leaf, measured in pressure units.	[43, 44]	[45]
	*Xylem water potential (Ψ*_x_ *)*	MPa (MJ/m^3^)	Potential energy of water in the xylem, in pressure units.		

*m* = Mass

^1^RWC_phys_ is related to θ and VWS through stem volume or leaf area but needs the turgid mass to be computed

^2^Only available using remote sensors in laboratory experiments [46], to our knowledge

^3^Note that, as we detail in Sect. "Assessing vegetation water content: Building a bridge between remote sensing and plant ecophysiology", RWC as defined in remote sensing literature (RWC_RS_) is actually a relative signal strength, not RWC of the plant as defined in ecophysiology (RWC_phys_). In RWC_RS_ maximum and minimum of VOD can be substituted by percentiles 95 and 5, respectively, to reduce the impact of extreme values (outliers)

^4^LFMC = [m_g_/(1- m_g_)]·100 [18]

**Table 2:** Large-scale LFMC datasets. All of them, except [48] and [49] are intercompared in this study

Product	Region	Period available (Period intercompared)	Sensors	Res.	Approach
[50]	Global	2001–2019 (depending on the region)	MODIS	0.05º	Applies RTMs to simulate spectra for grasslands, shrublands and forests. Then, LUT inversions by land cover minimize differences between simulated and satellite data to estimate LFMC.
[36]	Global	1997–2018 (depending on the region)	TMI, WindSat, AMSR-E, AMSR-2, MODIS	0.25º	VOD, LAI, and tree cover are found to be the best estimators of LFMC based on field-scale regressions. Then, random forests are applied to tailor the regression parameters and build LFMC maps.
[49]^1^	CONUS	2014–2017 (N/A)	MODIS	500 m.	One year of MODIS reflectance data, meteorological data, and field LFMC data are used in multi-modal, multi-temporal convolutional neural networks (CNN) to estimate LFMC.
[48] ^2^	CONUS	2019–2021 (N/A)	MODIS	1 km.	Field LFMC, MODIS reflectance, LST, soil saturation, ET, land use and topography are used to calibrate a random forest model for LFMC estimation.
[37]	West US	2015–2019 (Apr. 2015 – Dec. 2018)	Landsat 8 OLI, Sentinel-1	250 m.	Landsat reflectance and Sentinel-1 radar backscatter are used in a recurrent NN to estimate LFMC with help of auxiliary variables (e.g., soil textures or canopy height).
[35]	West US	2015–2018 (Apr. 2015 – Dec. 2018)	AMSR-2, SMAP, GEDI, Sentinel-1	0.25º	A semi-physical algorithm applies canopy height and radar backscatter to estimate VOD. Differences with satellite VOD are minimized to derive the dielectric constant of vegetation (ε). Then, LFMC is derived from ε using dielectric models. LFMC derived from X-band VOD is used in the comparison, since it is the most consistent with leaves and twigs *in* situ samples [35].
[51]	Australia	2001–2017 (2001–2017)	MODIS	500 m.	Applies RTMs to simulate spectra for grasslands, shrublands and forests. Then, LUT, land cover maps and a merit function are used to estimate LFMC.
[52]	Mediterranean basin	2001–2021 (Jan. 2001 – Jul. 2017)	MODIS	500 m.	Random forest models are built to estimate LFMC based on field LFMC, MODIS reflectance, vegetation indices, surface temperature and the day of the year.

^1^Since the product is not publicly available it was not included in the product comparison. ^2^The study period in [48] does not match the study period of other datasets in the region. Hence [48], is excluded from the comparison. Acronyms: *LUT* (look-up-table), *GEDI* (Global Ecosystem Dynamics Investigation), *TMI* (Tropical Rainfall Measuring Mission’s Microwave Imager)

Satellites allow regular monitoring of the land surface from local to global scales. They capture vegetation water dynamics through water-related reflectance changes with optical sensors [53], changes in leaf temperature with infrared sensors measuring thermal emissions [17], or water-induced changes in emission and backscatter with microwave sensors [18]. Importantly, different frequencies at which the sensors operate have different sensing depths within the canopy. Optical and thermal sensors, similarly to high frequency microwaves (e.g., Ku-band; ~18 GHz), are only sensitive to the top-of-the-canopy leaves, while low microwave frequencies (e.g., L-band at 1.4 GHz) are likely sensitive to the full canopy depth [54–57]. This frequency-depth relationship also depends on the canopy density and homogeneity [58]. Thus, the choice of the frequency and remote sensing technique to be used depends on the system and tissue that one aims to study. High microwave frequencies and some optical systems are appropriate for grasslands and shrublands and for forest leaf conditions, while low microwave frequencies (e.g., L-band) are appropriate to study the full forest canopy depth and are also sensitive to the woody tissues [54, 56].

Considering the spatiotemporal domain of satellite sensors, orbits of the satellites carrying optical and thermal sensors allow sub-weekly overpasses at spatial resolutions down to 10 m (or submeter scales for some commercial satellites) [59, 60]. However, the effective temporal availability of data can be reduced because these sensors cannot measure the surface when it is covered by clouds. Active microwave satellites have comparable spatial resolution of tens of meters but at mostly biweekly time resolutions (e.g., Sentinel-1, ALOS-2-PALSAR, or NISAR [61, 62]). Passive microwave satellite sensors such as those on the Soil Moisture and Ocean Salinity (SMOS; [63]), on the Soil Moisture Active-Passive (SMAP; [64]), and the Advanced Microwave Scanning Radiometers (AMSR-E/2/3; [65, 66]) have coarse resolutions (15–40 km) but higher temporal resolution (2–3 days). Unlike optical sensors, microwave remote sensing is largely unaffected by clouds.

Despite the recent advances in remote sensing for monitoring water in vegetation [35, 36, 42, 43] and the growth of *in situ* datasets across more locations [19, 41, 45], significant challenges remain in connecting remote sensing with *in situ* data characterizing the water status of plants. This is due to differences in spatial resolution (point-to-pixel scale gap) and to the need to improve the methods used to translate remote sensing observations into accurate and reliable indicators of plant water status. For instance, [24] details how satellite estimates of vegetation water storage (VWS) can provide information linked to the water status of plants, and highlights the need for upscaling field studies in ecophysiology from plot to pixel scales by developing pressure-volume curves [67] at the ecosystem scale (EPV curves) [68, 69]. These are water content vs. water potential relationships established beyond the tree level that can be applied to derive vegetation water potential (Ψ) information from satellite VWS estimates [24, 68, 69]. Also, [70] coins the term ‘water potential information gap’, describing the existing gap between sparse, irregular samples of *in situ* vegetation Ψ (mostly based on pressure chamber measurements) and the need for more regular and continuous data of this variable. This gap could be bridged with a more extensive use of psychrometers, by deploying Ψ networks like PSInet [45], and by linking them to the targeted but still unachieved large-scale Ψ estimation from remote sensing [70]. The remainder of this manuscript will investigate this and other research gaps in monitoring water in vegetation, including water content, fuel moisture, and water potential.

**Fig. 1:**
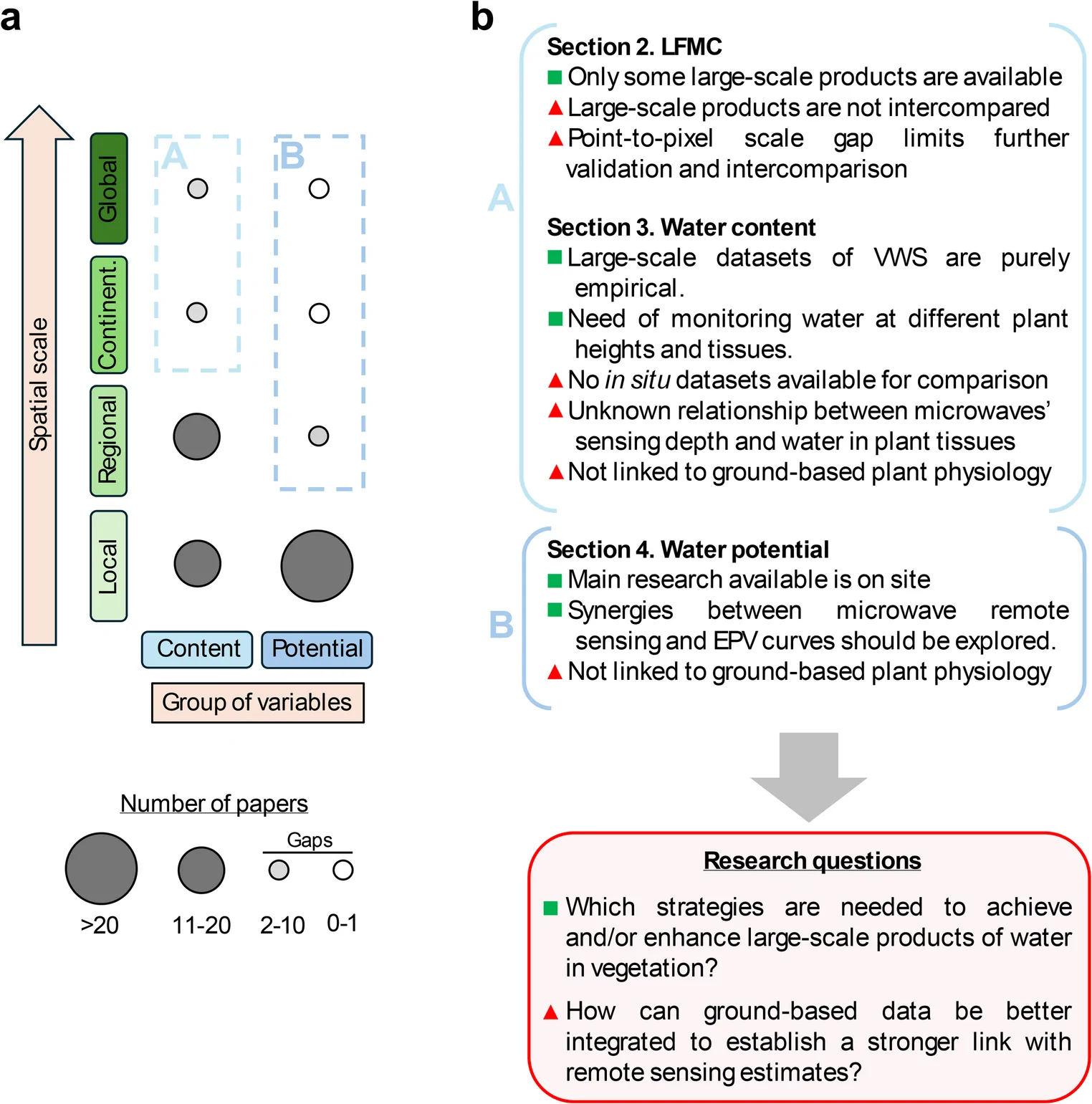
**a** Number of remote sensing papers selected ordered by spatial scale (rows) and by groups of variables in Table 1 (columns; ‘water content’ and ‘water potential’). Gaps are identified when ten or fewer papers are found in each row-column pair for the period 2014–2024. The list of literature references containing all the papers counted in this figure is in Supplementary References. **b** A text box for each group of variables details the main research gaps or challenges at large scales and the section in the paper where they are addressed. These challenges lead us to the two main research questions of our paper (red box). Questions and challenges are linked by green squares and red triangles in the figure. Note that “large scale” in Fig. 1b refers to continental and global scales. See SI1 for more details on the methodology of the review

To illustrate the main knowledge gaps, we conducted a structured literature search for the period 2014–2024 (see SI1 for the methodology) and summarized the results in Fig. 1. As a result, Fig. 1a classifies the most relevant literature on remote sensing of vegetation water content and water potential. We observe that, while local-scale research on water in vegetation is abundant, important gaps remain at regional to global scales (Fig. 1a).

We focus on large-scale gaps by exploring how regional to global remote sensing of the focal variables (Table 1) can be better linked with existing *in situ* datasets. Specific research gaps for each variable (Fig. 1b) are explored in Sects. "Monitoring live fuel moisture content (LFMC) " to "A road towards monitoring vegetation water potential (ψ) from remote sensing". Section "Monitoring live fuel moisture content (LFMC) " provides, to our knowledge, the first comparison among large-scale LFMC datasets and against *in situ* data (see [71] for one smaller-scale example). The same section analyses which strategies should be used to bridge the spatial-scale mismatch between *in situ* LFMC data and pixel-scale satellite estimates in validation procedures. Section "Assessing vegetation water content: Building a bridge between remote sensing and plant ecophysiology" addresses the challenges related to water content. This includes empirical simplified estimates of VWS [25], the lack of plot-scale data to validate them, and the need to understand which ecophysiological processes, canopy levels, and plant tissues are represented in satellite vegetation water estimates. Finally, Sect. "A road towards monitoring vegetation water potential (ψ) from remote sensing" reviews new opportunities based on passive microwave signals and multi-frequency sensor synergies that can enable the assessment of spatial, temporal and vertical variability of Ψ [44, 72].

In summary, the research gaps addressed in this study can be grouped into two main research questions. First, which strategies are needed to achieve and/or enhance large-scale products of water in vegetation? Second, how can ground-based data be better integrated to establish a stronger link with remote sensing estimates? (Fig. 1b). The goal of this paper is to address these questions and provide guidance on how the gaps illustrated in Fig. 1 can be reduced. This review intentionally pays special -although not exclusive- attention to passive microwave remote sensing for several reasons. First, passive microwaves are particularly appropriate to sense water across different canopy layers and have demonstrated large potential for the assessment of vegetation water status [18, 24]. Second, plot-scale microwave-based approaches relying on Global Navigation Satellite Systems (GNSS) [73, 74] are expanding and will complement *in situ* samples for validation and interpretation of microwave satellite vegetation water estimates. Third, intensive research has been conducted in the passive microwave remote sensing field on the connection between satellite and GNSS measurements with vegetation canopy water variables during the last five years. We review this research and propose further development.

This review aims to inform a wide range of research fields and end-users working across scales, including plant ecophysiologists, fire risk assessment agencies (mainly working with LFMC data), and Earth system modelers (e.g., using vegetation water status to improve land-atmosphere feedback estimates). Our work is applicable to all vegetation types, even if some discussion focuses on forests, where the availability of different microwave frequencies has the advantage of being sensitive to different canopy layers and tissues.

## Monitoring Live Fuel Moisture Content (LFMC)

### LFMC Estimates from Remote Sensing: Physical Background and Available Products

LFMC refers to the mass of water contained in living plant matter, expressed relative to its dry mass. Historically, the optical domain has been used to infer LFMC. Water in plants mainly absorbs the electromagnetic waves in the short-wave infrared (SWIR) and, to a lesser extent, in the near infrared (NIR) regions of the spectrum. Thus, variations in absolute water content in plants modify the NIR and SWIR reflectances, leading to covariation between water content and NIR/SWIR [17, 53]. In addition to this direct effect, plant water stress can also indirectly influence reflectance through the depletion of chlorophyll content, which affects the visible region of the spectrum. LFMC retrievals from optical sensors generally rely on vegetation indices and statistical methods [75–78], machine learning [48, 49, 52, 79–81], or the inversion of radiative transfer models (RTM) [50, 51, 82–85]. In the review period (2014–2024), six large-scale LFMC estimates have been published using only optical sensors [48–52]. These products are listed and briefly described in Table 2.

The microwave domain of the spectrum is also sensitive to changes in the water content of plants, since water modifies the dielectric constant of the canopy, and thus its capacity to emit, reflect and attenuate electromagnetic waves [18]. Because of this, data from microwave sensors are used to estimate the vegetation optical depth (VOD), a variable quantifying the attenuation of the microwaves that pass through vegetation after being emitted by the land surface. VOD is not only sensitive to the water, but also to the biomass and structure of different parts of the canopy, thus being high for wet and dense canopies [86]. Recent research has isolated the water component (expressing it as LFMC) from the biomass-structure component of the VOD signal. It has done so by applying semi-physical algorithms using multi-sensor synergies both in the western USA [35] or globally [87]. Also, large-scale LFMC products have been established by combining Sentinel-1 radar backscatter with Landsat 8 reflectance in the western USA [37], or have used statistical approaches combining VOD and leaf area index (LAI) to map LFMC globally [36] (Table 2).

### Field Data Observations of LFMC

The most comprehensive *in situ* LFMC dataset has been gathered in the Globe-LFMC database [41], which has been recently updated in 2024 (Globe-LFMC v2; [19]). The updated version contains ~ 294,000 LFMC samples from 2,245 sites collected across 15 countries over the period 1977–2023. However, it is worth noting that 80% of data and 55% of sites are in the United States.

*In situ* LFMC data is measured in single leaves (only occasionally in small twigs), usually at the top of the canopy, and most samples are taken during the summer [19, 41]. These measurements are destructive and labour intensive. LFMC is an appropriate variable to predict the risk of flammability and fire propagation, especially in short plants, since the leaves’ moisture is representative of the entire plant hydric conditions. In the case of forests, LFMC cannot represent the water status of entire trees, since it is not measured in stems or branches along the full trees’ heights. Still, it accounts for the dryness of tree crowns and of the understory, which are main vectors in fire propagation.

The global availability of *in situ* LFMC has two main advantages for scientific research and satellite product development. First, the large amount of data is an essential source of information for machine learning approaches to produce more robust large-scale satellite LFMC estimates. Indeed, MODIS and Globe-LFMC have been combined to build LFMC maps across both the continental USA [48, 81] and the Mediterranean basin [52]. Secondly, Globe-LFMC has also been used as benchmark data to validate large-scale satellite-derived LFMC datasets [35, 36, 52, 81]. However, each of these products has been validated individually, and limited effort has been put into using global *in situ* LFMC data to compare them (one example is [71], but it is confined to Israel). Consequently, we have limited knowledge of how the different products and their underlying techniques perform comparatively. We aim to close this gap by conducting filtering and comparison analyses in the next section.

### Comparison of LFMC Datasets and Assessment of the Point-to-Pixel Scale Mismatch

A central obstacle in evaluating LFMC products is that the field measurement and the satellite retrieval do not represent the same spatial scales of the vegetation components. They also represent different components of the canopy: field LFMC is usually measured on selected leaves or small twigs, whereas satellite products integrate over mixed pixels and, depending on the sensor, over different canopy components. We therefore use the available product comparison not only to rank datasets, but to identify which kinds of filtering improve the biological representativeness of validation sites.

The literature review finds eight datasets published in the period 2014–2024, of which two are global, two span the entire conterminous United States (CONUS), two cover the western USA, and one each in the Mediterranean basin and Australia (Table 2). We will compare them against *in* situ data and explore the best quality filtering approaches in this section. We acknowledge that some of these products [36, 48, 51] have been calibrated using *in situ* LFMC data. This may impact the results by increasing correlations of these satellite products with *in situ* LFMC and by decreasing the error and bias statistics. Note that it is not possible for us to distinguish which sites were applied to the calibration (and, consequently, it is not feasible to discard them in the comparison).

The validation and comparison of LFMC estimates using *in situ* data is hampered by representativeness issues. There exists a point-to-pixel spatial scale mismatch, which, in the case of LFMC, refers to the capacity of a single point measurement to describe the moisture state and dynamics of plants in a pixel tens of meters to kilometers across that can be heterogeneous usually in terms of land cover (e.g., [35, 36]), but also in terms of the species sampled or in terms of the physical representativeness of the individual sample within a canopy [35, 41]. To deal with these issues, different filters exist to select the most representative sites for validation using, for example, the spatial variability of NDVI, the land cover heterogeneity in a satellite pixel, or metrics to determine the diversity of species sampled across a site (Table 3). To compare filters and products, we use the standard metrics Pearson’s correlation coefficient (r), RMSE and bias. Correlation is only meaningful when the *in situ* LFMC time series has sufficient temporal variability ([35, 37], Std_site_ in Table 3, and SI2).

**Table 3:** Homogeneity and quality filters reported in the literature which impact the validation results. In the column “Type” the spatial filters screen out pixels or sites with large spatial heterogeneity, the species filters aim at ensuring consistency between the plant species measured at a site and the dominant vegetation within a pixel, and the temporal filter screens out sites with low time-variability of *in situ* LFMC. Columns “More strict” and “Less strict” indicate the range of conditions studied in Fig. 2 for each filter

Name	Type	Description	Literature values	More strict	Less strict
CV_NDVI, Max_	Spatial	Maximum Coefficient of Variation (CV; [%]) in time of monthly averaged NDVI, computed on an area of 500 m × 500 m around the site’s coordinates, provided by [41]^1^.	15–30% [50] 26% [36] 25% [35]	15%	40%
D_LC_	Spatial	Land cover distance between 1 km and 0.25° pixels. It uses the percentages of tree cover (TC) and short vegetation (SV) from [88] at both resolutions: $\:{D}_{LC}=\sqrt{{\left({TC}_{25km}-{TC}_{1km}\right)}^{2}+{\left({SV}_{25km}-{SV}_{1km}\right)}^{2}}$	< 10% [36]	5%	20%
Sp1	Species	Exclude multi-species LFMC sites unless species LFMC time series are correlated (Pearson *r* ≥ 0.5).	[37]	Applied^2^	Not applied
Sp2	Species/ Spatial	Sites where x% of the sampled species are of the same vegetation type (tree, shrub or grass) as the dominant in the pixel.	100% [36]	100%	0%
LC	Spatial	Sites where the dominant land cover spans through ≥ x% of the pixel area are chosen	60% [35]	100%	0%
Std_site_	Temporal	Sites where standard deviation of LFMC in time is > x are chosen. In sites with low variability, correlation is less useful because variability can be driven by noise [35, 37]. This metric is applied in [35] but originally based on [37], where the authors applied the coefficient of variation in time (instead of the standard deviation).	-	0%	≥ 50

^1^Not available in Globe-LFMC v2, so we use Globe-LFMC v1.^2^We also test excluding all sites with > 1 species (regardless of correlations). Seasonalities are omitted due to poor information on how the authors built them

**Fig. 2:**
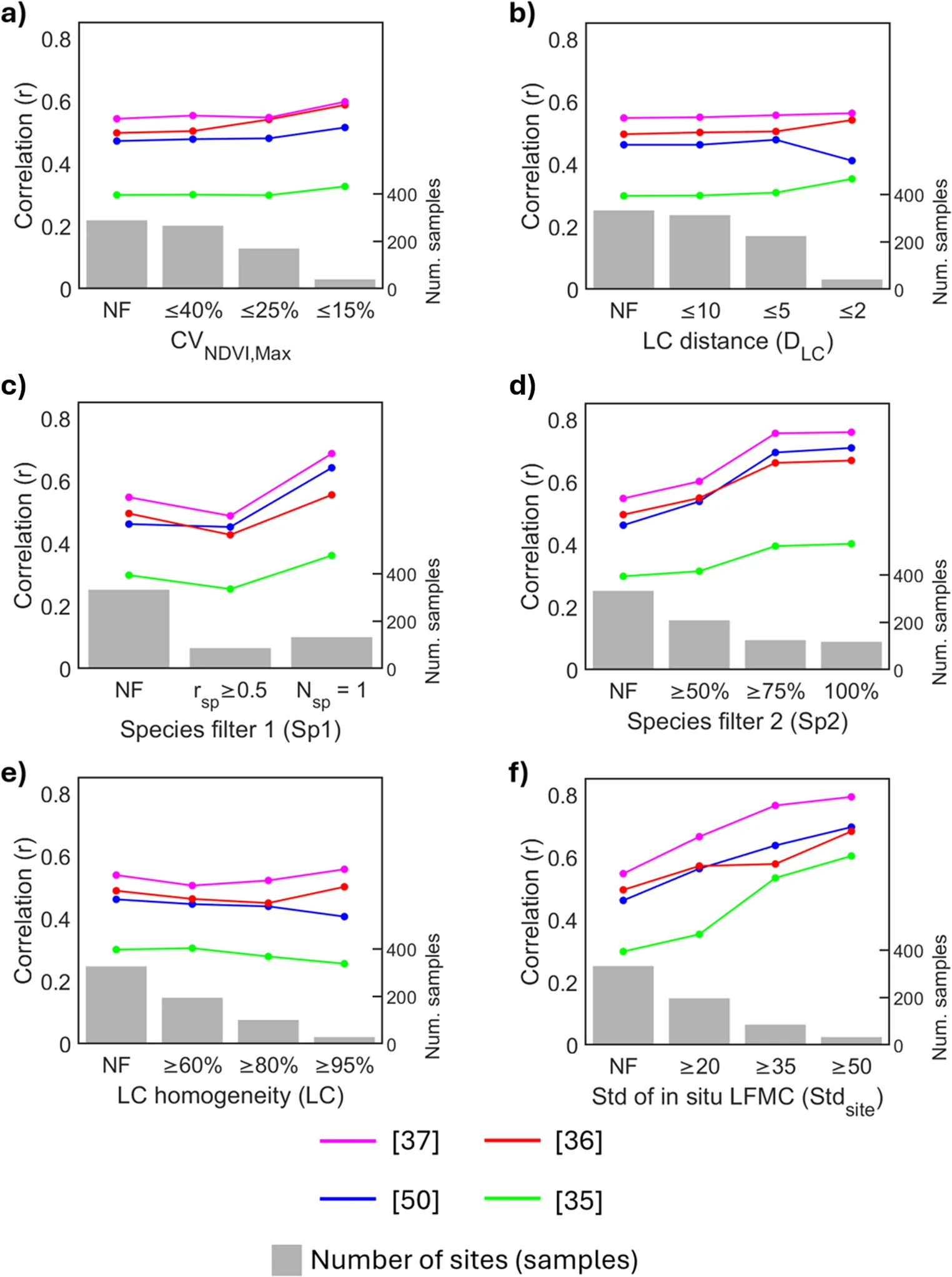
Western USA. Effect of progressively stricter site-screening criteria (as in Table 3) on the agreement (median correlation) between satellite-derived LFMC products and *in situ* LFMC measurements. The satellite LFMC products are represented by coloured lines. The legend provides the products’ references. The subfigures show: **a** the maximum spatial coefficient of variation of NDVI at a site (CV_NDVI, Max_), **b** the land cover ‘distance’ between 1-km and 0.25º pixels (D_LC_), **c** the filter removing LFMC sites with more than one species sampled (Sp1), **d** the percentage of sampled species which are of the same vegetation type as the dominant in the pixel (Sp2), **e** land cover homogeneity as the percentage of dominant land cover in the pixel (LC) and **f** standard deviation of *in situ* LFMC (Std_site_)

Here, we first evaluate how homogeneity and quality filters reported in the literature (Table 3) affect the comparison statistics of selected large-scale LFMC datasets (Table 2) in the western USA and the Mediterranean Basin (Figure S1). The SI2 provides methodological details. Correlations between remote sensing and *in situ* datasets in the western USA responded differently depending on the filter applied (Fig. 2). Filters based on land cover homogeneity (D_LC_ and LC) or vegetation greenness (CV_NDVI, Max_) generally had minimal effects, with correlation changes either absent or small (Δr < + 0.1). In contrast, filters representing the homogeneity of sampled species (Sp1 and Sp2) consistently led to stronger improvements, with correlations increasing by roughly 0.1 to 0.3 (Fig. 2c and d). These filters ensure that the species sampled are always the same or at least consistent with the pixel-dominant vegetation type. Future research should explore the impact of intra-specific variability across climate, soils and topography, which are relevant to explain moisture variations in plants [89]. Also, it is important to consider the variability in time of the *in situ* data. As expected, sites with high Std_site_ show a higher correlation than sites in which time-variability of *in situ* LFMC is low (Fig. 2f). Generally, the results presented are consistent with those obtained in the Mediterranean basin (Figure S2) and show that three filters are promising to achieve consistent screening across products and regions: Sp1, Sp2 and Std_site_ (see Table 3 for more details on each filter). Still, in some cases, applying filters Sp1 and/or Std_site_≥35% increases the bias and/or the RMSE (Figures S3 to S6), suggesting that Sp1 may not be fully appropriate and that a lower Std_site_ of 20% enhances the selection criteria.

Combining the species homogeneity or time variability filters with land cover ones does not change the results significantly (not shown). This confirms how accounting for land cover homogeneity is not enough to bridge the point-to-pixel scale mismatch. This representativity issue is particularly important in short-statured, agricultural, and mixed shrub–grass systems. In such ecosystems, water content and water potential can often be measured directly using destructive sampling and pressure-chamber measurements. Therefore, the main limitation is not necessarily the feasibility of field measurements, but whether those measurements represent the vegetation component sensed by the satellite pixel. For example, coarse pixels may include mixtures of bare soil, litter, grasses, shrubs, crops, irrigated areas, or vegetation at different phenological stages. In low-biomass systems, the vegetation water signal can also be more strongly affected by soil background, surface wetting, canopy cover, and management practices than in closed forests. Future research should therefore combine LFMC or water-potential measurements with information on fractional vegetation cover, species or crop type, phenological stage, management or irrigation status where relevant, and repeated sampling across the satellite footprint.

**Fig. 3:**
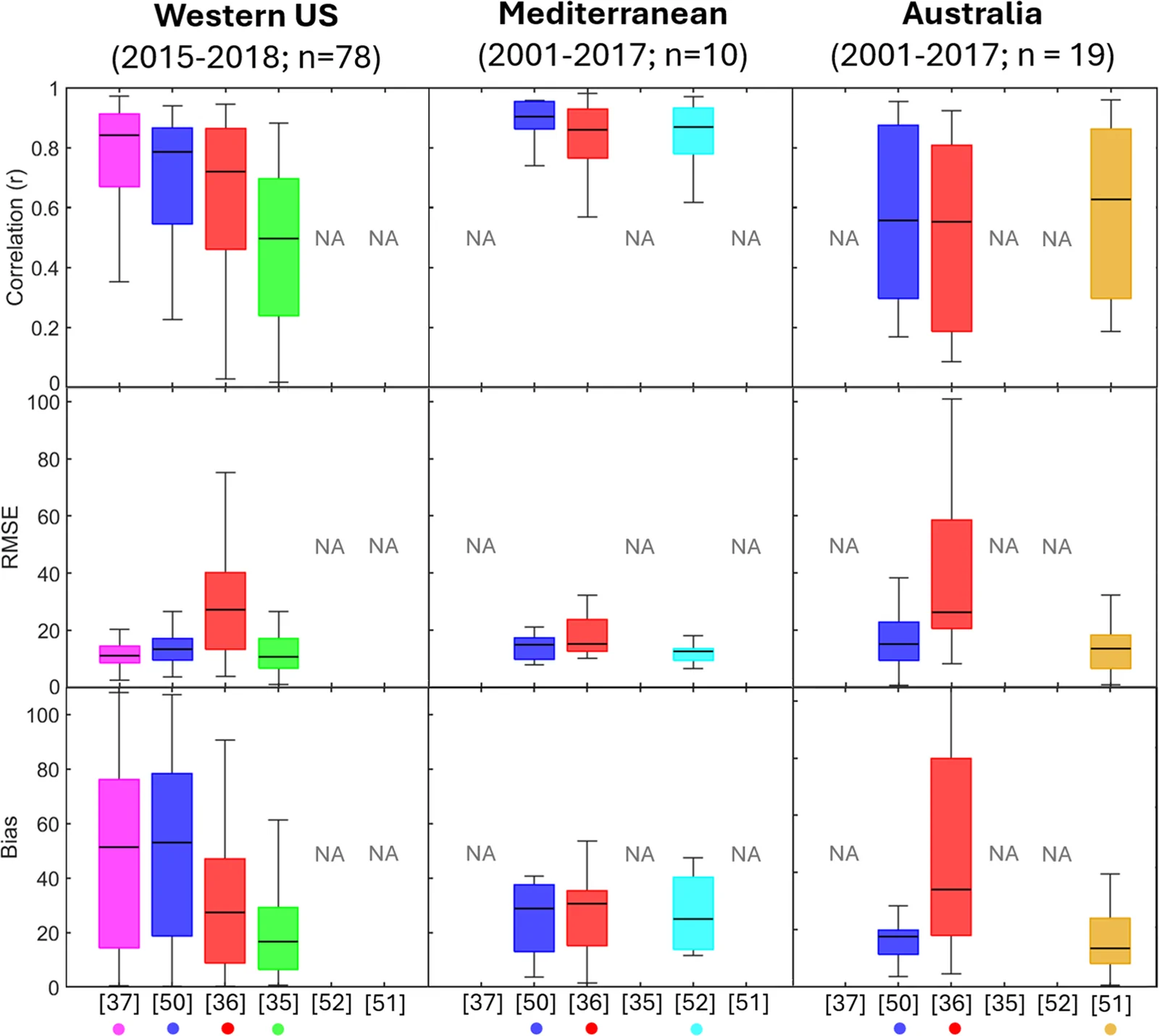
Comparison between satellite and *in* situ LFMC time series. Columns show the regions; rows correspond to temporal correlation, RMSE, and bias. Boxplots represent median (black line), 25th and 75th percentiles (limits of the boxes) and 5th and 95th percentiles (whiskers)

In a second step, we undertake our comparison among LFMC products in the Western USA, the Mediterranean, and Australia using the best filtering strategy identified earlier (Figure S1 and Table S3). According to the results on filters, we simultaneously apply filters Sp2 = 75% and Std_site_≥20% and discard other combinations (SI2). Overall agreement between the products and the *in situ* samples was moderate to strong across the three regions (median r: 0.5–0.9, RMSE < 30%, median bias < 60%; Fig. 3). Note that the RMSE and bias percentages are in LFMC units, which means that, for example, a bias of 60% represents 15% of the maximum range of LFMC. This is based on the fact that LFMC can reach ~ 400% according to [19] and assumes a minimum LFMC of zero.

In the western USA, three products have high median correlations, ranging from 0.72 to 0.84, while the product in [35] shows a smaller median correlation (*r* = 0.5), but a lower bias (< 20%). We suggest that this lower correlation in time may be due to the different timescales used in the dynamic parameters (δ and VOD) applied in the semi-physical model used in [35]. A low bias is also found in the region in [36]. Low biases in both VOD-based products suggest that accounting for vegetation structure using LAI [36] or removing the biomass and structure signals in VOD using canopy height and radar data [35] can isolate the vegetation moisture component from the passive microwave signal. Products based on optical combined with radar data [37] or without it [50] show the highest correlations in the western USA and keep the median *r* ~ 0.8 in the Mediterranean, like the product in [52]. This might be influenced by the higher original spatial resolution of optical products compared to that of the VOD-based ones, even if an aggregation has been conducted (SI2).

Results in Australia and the Mediterranean show that the product in [36] (passive microwaves) and optical products [50, 51] are similar in correlation. The bias and RMSE in Australia are low for [51] and [50] which, together with their high resolution, make these products suitable for LFMC estimations there. The correlations in Australia (0.55 < *r* < 0.63) compared to those obtained in the Mediterranean basin and the western USA (0.72 < *r* < 0.90 for four out of five products) would likely be greater if quality filters could have been applied. Finally, it is worth noting that we have not found a specific technique (i.e., random forests, neural networks, RTMs, or semi-physical algorithms) outperforming others in the estimation of LFMC (see Fig. 3 and Table 2).

## Assessing Vegetation Water Content: Building a Bridge Between Remote Sensing and Plant Ecophysiology

### Monitoring the Water Status of Vegetation

While LFMC is principally applied in fire risk assessment, water in an ecosystem can also be measured using variables that represent the water status of plants and are directly linked with ecosystem functions including the fluxes of water, carbon and energy. The vegetation water storage (VWS) [$\:\frac{{kg}_{water}}{{m}_{ground}^{2}}$] is the water contained in the vegetation relative to ground area. It is directly convertible to the absolute mass of water when multiplied by the area (e.g., pixel size), or to the water column when divided by the water density. The latter is also named equivalent water thickness (EWT [mm]; Table 1). VWS or EWT are extensive variables (i.e., variables depending on the size of the system; [68]) which scale linearly with biomass. Generally, a high-biomass forest contains more water and thus a larger VWS than a similarly hydrated forest with less biomass. On the other hand, water content relative to wood volume (θ_stem_; [$\:\frac{{m}_{water}^{3}}{{m}_{wood}^{3}}$]) and water content relative to leaf area (θ_leaf_; [$\:\frac{{kg}_{water}}{{m}_{leaf}^{2}}$]) are intensive variables (i.e., independent of the size of the system) representing physical properties of the tissues (wood and leaves) [68]. Ecophysiologists also define water content relative to a tissue-level saturated value (RWC_phys_) (Table 1). θ and RWC_phys_ are used in ecophysiological field research -together with Ψ- to study the plant water status and its vulnerability to drought [67].

Currently, satellite-based mapping of water content relies mostly on estimating the VWS (e.g., microwave measurements are more sensitive to VWS than to intensive variables). This limits our ability to fully quantify water status of forests independently of the size of the system, as needed in ecophysiological research. To our knowledge, remote sensing of θ_stem_ and θ_leaves_ has only been conducted in laboratory experiments using terahertz spectroscopy, which is not comparable to satellite techniques [46]. The lack of direct measures of θ is a clear limitation to enable monitoring of forest water status from space.

RWC, and its approximation in the remote sensing community (here called RWC_RS_), is poorly related to the ecophysiological meaning and definition of RWC_phys_. RWC_RS_ is computed by normalizing the sensor signal (e.g., VOD) between percentiles 5 and 95 of the time-series, usually considering periods of relatively stable biomass to ensure that changes in VOD are only due to variations in the water content [24, 32, 33, 43, 72]. Thus, it represents a fractional variation within the sensed range of values, that is, a relative measure of signal strength. An RWC in the ecophysiological sense would require remote observations to determine when the vegetation is at the saturation point and when it is completely dry (see the RWC formulas in Table 1). Importantly, RWC_RS_, which varies between 0% and 100%, is different in range from RWC_phys_, which typically varies between > 70% and < 100%. This minimum value of ~ 70% is explained by mortality thresholds in woody tissues being around 70% to 90% of the saturated value [90] and has considerable variation. Moreover, the range obtained by RWC_RS_ is dependent on the conditions during the measurement period (e.g., the lowest/highest values in the remote sensing time-series are assumed to be representative of extreme drought/wetness situations, which may not be the case). Still, RWC_RS_ has been used to explain regional-scale forest mortality [32] and has been related to Ψ (Sect. "A road towards monitoring vegetation water potential (ψ) from remote sensing"). This can be explained because RWC_RS_ and RWC_phys_ can be proportional and because normalizing a remote sensing signal like VOD allows comparison of different ecosystems across space. Notwithstanding, there exists a relevant conceptual and naming gap between RWC_phys_ and RWC_RS_.

**Fig. 4:**
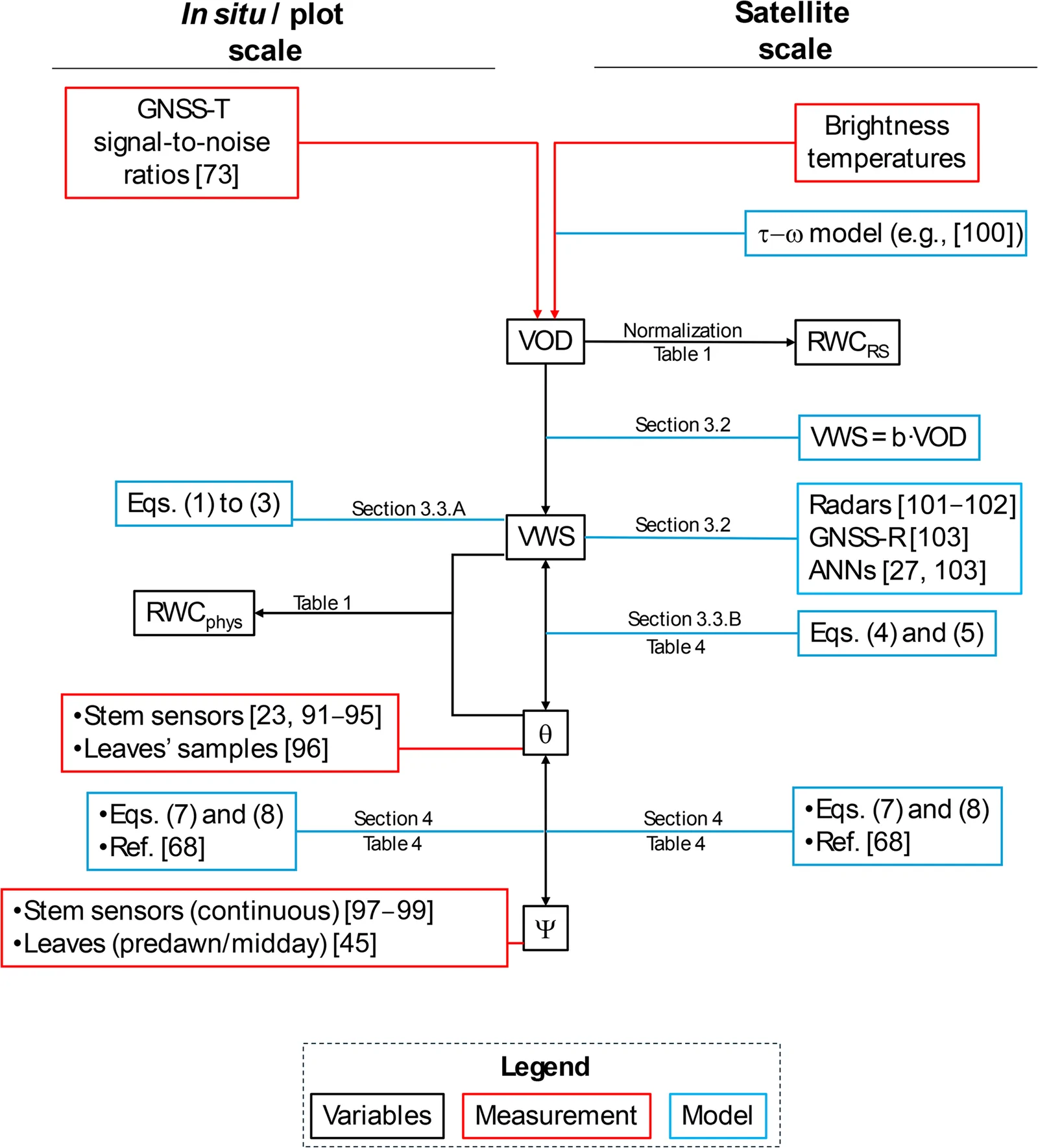
Workflow linking VOD estimations with the plant water status through VWS at *in situ* and plot scales (left side) and at satellite (coarse) scale (right side). Black arrows and boxes indicate variables and the fluxes between them. Red lines and boxes indicate measurements, while blue ones indicate models. Sections and tables where each step is described are referred above or below these lines. The workflow shows how VOD is retrieved from satellites using the τ−ω model (or at the plot level using GNSS-T). VWS is then retrieved from satellite VOD using the b-parameter approach, but alternative techniques at satellite (e.g., GNSS-R) and plot (Eq. (1) to (3)) scales have been proposed. We show how VWS and Ψ are related via θ by using EPV curves [68], which can theoretically allow Ψ estimations at both plot and satellite scales. Both θ and Ψ can be measured *in situ* for validation purposes or, in the case of Ψ, estimated with vegetation models. The workflow also shows that RWC_phys_ can be derived from VWS or θ, and that normalizing VOD leads to a remote-sensing-based proxy of (but not an equivalent quantity of) RWC (i.e., RWC_RS_). Note that LFMC is not included in the workflow for the sake of simplicity (but check Eqs. (3), (5) and (6) in the text)

Still, estimating VWS, RWC or θ alone is not enough for monitoring the water status of vegetation. This is because: (i) plant growth, ecosystem dynamics, and carbon and water cycles are mediated by Ψ, (ii) pressure-volume curves, which define the relationship between θ (or RWC_phys_) and Ψ, have not been established beyond the tissue scale (i.e., leaves and sapwood), and (iii) these relationships likely vary along the vertical profile of plants [24]. These are relevant gaps between large-scale remote sensing and the current local-scale capacity to assess the water status of vegetation in ecophysiology, which is almost exclusively based on field data. To help bridge this gap we propose a workflow to derive not only water content, but also water potential, from remote sensing VOD data (Fig. 4). In the following sections, we review the current satellite VWS research (Sect. "Mapping VWS from space") and address the gaps pointing to the potential of developing satellite θ measurements (Sect. "Linking satellite and *in situ* water content information"), the relevance of vertical gradients (Sect. "Relationship of microwave sensing depth to the water status from different plant heights and tissues"), and how satellite-derived Ψ estimates can be approached (Sect. "A road towards monitoring vegetation water potential (ψ) from remote sensing").

### Mapping VWS from Space

Top-of-canopy VWS (i.e., CWS [$\:\frac{{kg}_{water}}{{m}_{ground}^{2}}$]; Table 1) has been retrieved by establishing a relationship with satellite optical reflectances using RTMs and machine learning [30, 31]. Also, VWS has been estimated based on NDVI and stem factors (i.e., the product between the average height of a land cover class and the ratio of sapwood to leaf area [104]). In both cases, the use of optical data constrains the sensing depth to top-canopy leaves and branches, at least in closed canopies. With these approaches, dryness in canopies can be detected, but it cannot be understood whether it is caused by (i) steep vertical gradients of water status (i.e., dry leaves but wet stems, which is common during periods of high transpiration) or (ii) entirely dry vegetation under stress, reaching critical conditions.

Passive microwave remote sensing uses the linear relationship between VOD and VWS to estimate VWS. This direct relationship is enabled by a constant called ‘*b-parameter’* [86] (Fig. 4). However, the values of the *b-parameter* are generally assumed to be time-invariant and mostly based on a land cover look-up table [104]. The *b-parameter* fields have limited spatial variability and do not consider phenological changes [105, 106]. Consequently, they are subject to uncertainty and debate in the microwave retrieval algorithm community [106, 107]. Thus, a better understanding of the spatio-temporal variation of the *b-parameter* is needed to propose enhanced formulations to retrieve it. Alternatively, semi-empirical models and artificial neural networks (ANNs) based on multi-frequency microwave remote sensing provide good results when estimating VWS at high microwave frequencies [27]. VWS has also been mapped with radars [101, 102] and with global navigation satellite systems reflectometry (GNSS-R) measurements, for instance from the CYGNSS satellite [103] (Fig. 4). In fact, CYGNSS was used to estimate tropical and sub-tropical VWS using linear models and ANNs, being the only quasi-global-scale VWS map based on L-band data which does not use the *b-parameter* [103]. This suggests an interesting complementarity of AI with new signals of opportunity to monitor VWS.

### Linking Satellite and *In situ* Water Content Information

#### A. Building Plot-Scale VWS Estimates to Validate and Interpret Satellite VWS

The main limitation of satellite-based VWS estimates is that independent data for validation is lacking. *In situ* VWS measurements have been published only in few cases (e.g. [29]). Meanwhile, the US National Ecological Observatory Network (NEON) database contains canopy water indices obtained from airborne optical reflectance measurements [108]. Also, recent developments have led to global navigation satellite systems transmissometry (GNSS-T) sensors enabling plot-scale (up to ~ 100 m in diameter) estimates of VOD. These estimates are based on the difference in signal-to-noise ratios between antennas placed above and below the canopy [73]. However, these are still indirect estimates of local-scale VWS, not retrievals of it. This lack of point-to-plot scale VWS data also limits our understanding of plant-water relations across scales. To bridge this gap, a potential approach can be the estimation of plot-scale VWS derived from GNSS-T VOD data, which has already been used to estimate vegetation moisture [73] (Fig. 4).

Here, we also propose another approach taking advantage of *in situ* measurements of θ_stem_ and θ_leaf_ available from ecophysiological research sites [23, 95, 96]. These measurements have to be upscaled and transformed into plot-scale VWS estimates, which can serve for validation of satellite VWS or CWS (Table 1; Fig. 4) if the vegetation in the plot is representative of that in the pixel (Sect. "Monitoring live fuel moisture content (LFMC) ").

The purpose of the following equations is not to propose a single operational retrieval algorithm, but to explicitly state which field measurements are needed to connect tissue-scale water content with plot- or pixel-scale VWS. Stem and leaf water stores require different scaling approaches. Thus, for stems (adapted from [68]):

1 $${VWS}_{stem}=\frac{\sum_{i=1}^{i\:sp}{\stackrel-{\theta}}_{stem,i}.{\rho}_{water}.{V}_{wood,i}}{{A}_{ground}}$$

where$\:{\stackrel{-}{\:\theta\:}}_{stem,i}$ is the average θ_stem_ measured *in situ* for each * i* species, $\:{\rho\:}_{water}$ is the density of liquid water [1000 kg/m^3^], $\:{A}_{ground}$ is the plot ground area [m^2^] and $\:{V}_{wood,i}$ is the volume of wood [$\:{m}_{wood}^{3}$] of each species. Note that θ_stem_ can be measured by ecophysiologists using stem sensors based on time- or frequency-domain reflectometry [109] or electric resistance sensors [99]. Networks of such sensors can become a reality soon since there has been progress in developing general standards for their calibration [110]. Plot-scale $\:{VWS}_{stem}$ can be used for validation and interpretation of VWS derived from L-band VOD because: (i) L-band has large canopy sensing depth, and (ii) its long wavelength (~ 21 cm) makes it much more sensitive to larger structural components (stems, branches, trunks) than to leaves (see Sect. "A road towards monitoring vegetation water potential (ψ) from remote sensing" and [111]). Still, note that reliably deconvoluting the contributions of leaves and stems within the L-band signal is not possible. We acknowledge this as an inherent limitation of this approach.

To estimate plot-level VWS in leaves, one has to use Eq. (2) (adapted from [68]):

2 $${VWS}_{leaves}=LAI.\sum_{i=1}^{i\:sp}{\stackrel{-}{\theta}}_{leaf,i}\cdot{A}_{f,i}$$

where * LAI* is the leaf area index [$\:{m}_{leaves}^{2}$/$\:{m}_{ground}^{2}$], $\:{\stackrel{-}{\:\theta\:}}_{leaf,i}$ is the average θ_leaf_ measured *in situ* for each * i* species and $\:{A}_{f,i}$ is the leaf area fraction covered by each * i* species in the plot. Importantly, plot-scale * LAI* and leaf area fractions, as well as $\:{V}_{wood}$ in Eq. (1), can be estimated with terrestrial laser scanners (TLS) [112–114] or UAV-based LiDAR sensors [115]. In cases where the plot under study is sufficiently representative, plot-scale $\:{VWS}_{leaves}$ can be used for validation and interpretation of satellite CWS derived from high VOD microwave frequencies [27] (X- and Ku-bands; Sect. "A road towards monitoring vegetation water potential (ψ) from remote sensing") or from optical satellite sensors [30]. In addition, they can be used to compare plot-scale VOD and derived estimates based on GNSS-T sensors [73] (Fig. 4).

An alternative for the estimation of $\:{VWS}_{leaves}$, in this case at the plant scale, can be using data from *in situ* LFMC networks, which are mostly based on samples of leafy tissues (only occasionally of small twigs) [19, 41]. $\:{VWS}_{leaves}$ and LFMC can be linked using *in situ* estimates of LAI and specific leaf area (SLA; [m^2^_leaf area_/kg_leaf dry biomass_]) [116], as shown in Eq. (3):

3 $${VWS}_{leaves}={\theta}_{leaf}\cdot LAI=\frac{\left(\frac{LFMC}{100}\right)\cdot LAI}{SLA}$$

Since SLA and LAI are not contained in LFMC network databases, we suggest adding them in future versions as linking variables between the research fields of remote sensing, fire risk assessment, and plant ecophysiology. Note that (3) will be applied using individual LFMC measures, and experimental SLA and LAI estimates, not variables at plot-aggregated scales as in (1) and (2). Still, we acknowledge the fact that LAI and SLA are variables that can be measured using different techniques in the field, such as direct destructive methods or indirect optical methods (e.g., plant canopy analyzer LAI-2000). Sampling issues and assumptions for the optical methods may be especially relevant in short vegetation systems due to small leaves. To partially overcome these limitations in our approach, we suggest using the standardized methods for ground LAI and SLA measurements which are defined in the TRY database [34]. In these systems, validation should also account for fractional cover, phenological stage, exposed soil, and management practices such as irrigation or harvest, because these factors affect the relationship between field samples and the remotely sensed pixel. Also, we note that Eqs. (2) and (3) are the ones to be used in grasslands and croplands.

#### B. Towards estimating $\:\theta\:$ from satellite remote sensing

Rearranging Eq. (1, 2 and 3) also provides potential frameworks for estimating $\:{\theta\:}_{stem}$ and $\:{\theta\:}_{leaf}$ from space (Fig. 4). For stems, L-band VOD-derived VWS ($\:{VWS}_{LVOD}$) has to be used, although we acknowledge again the impossibility of fully isolating the stems’ signal from that of leaf tissues within satellite observations, and the simplification of basing VWS estimates on the *b-parameter* approach (Sect. "Mapping VWS from space"). To estimate $\:{\theta\:}_{stem}$ it has to be isolated from (1):

4 $${\theta}_{stem}=\frac{{VWS}_{LVOD}\cdot{A}_{pixel}}{{V}_{wood}\cdot{\rho}_{water}}=\frac{{VWS}_{LVOD}\cdot{A}_{pixel}\cdot{\rho}_{wood}}{{\rho}_{water}\cdot{B}_{wood}}$$

This approach can use large-scale maps of: (i) wood density ($\:{\rho\:}_{wood}$) which is modeled globally [117], (ii) current [25] or enhanced L-band VWS maps, and (iii) wood biomass ($\:{B}_{wood}$) which can be obtained from global above-ground biomass maps (e.g. [118]), assuming that woody biomass largely dominates over leafy biomass (Table 4). Since this assumption is held in woody systems [125] and $\:{\rho\:}_{wood}$ maps are only for woody vegetation [117], the $\:{\theta\:}_{stem}$ estimates will be here limited to woody plants as well.

$\:{\theta\:}_{leaves}$ can eventually be obtained from (2) and (3):

5 $$\:{\theta\:}_{leaves}=\frac{VWS}{LAI}=\frac{LFMC/100}{SLA}$$

LAI maps are commonly available from a range of multispectral satellite sources (e.g. [119]), and VWS for the top of the canopy (CWS) can be derived from optical remote sensing [30] or high-frequency VOD [27]. Also, LFMC (Table 2) and satellite-based SLA maps [120] can be used (Table 4).

Finally, by using L-band VOD-based LFMC ($\:{LFMC}_{LVOD}$) based on the approximation of [35], $\:{\theta\:}_{stem}$ can also be approximated as in (6):

6 $${\theta}_{stem}=\frac{\left({LFMC}_{LVOD}/100\right).{\rho}_{wood}}{{\rho}_{water}}$$

### Relationship of Microwave Sensing Depth to the Water Status from Different Plant Heights and Tissues

#### The Vertical Water Potential Gradient in the Forest Canopy

**Fig. 5:**
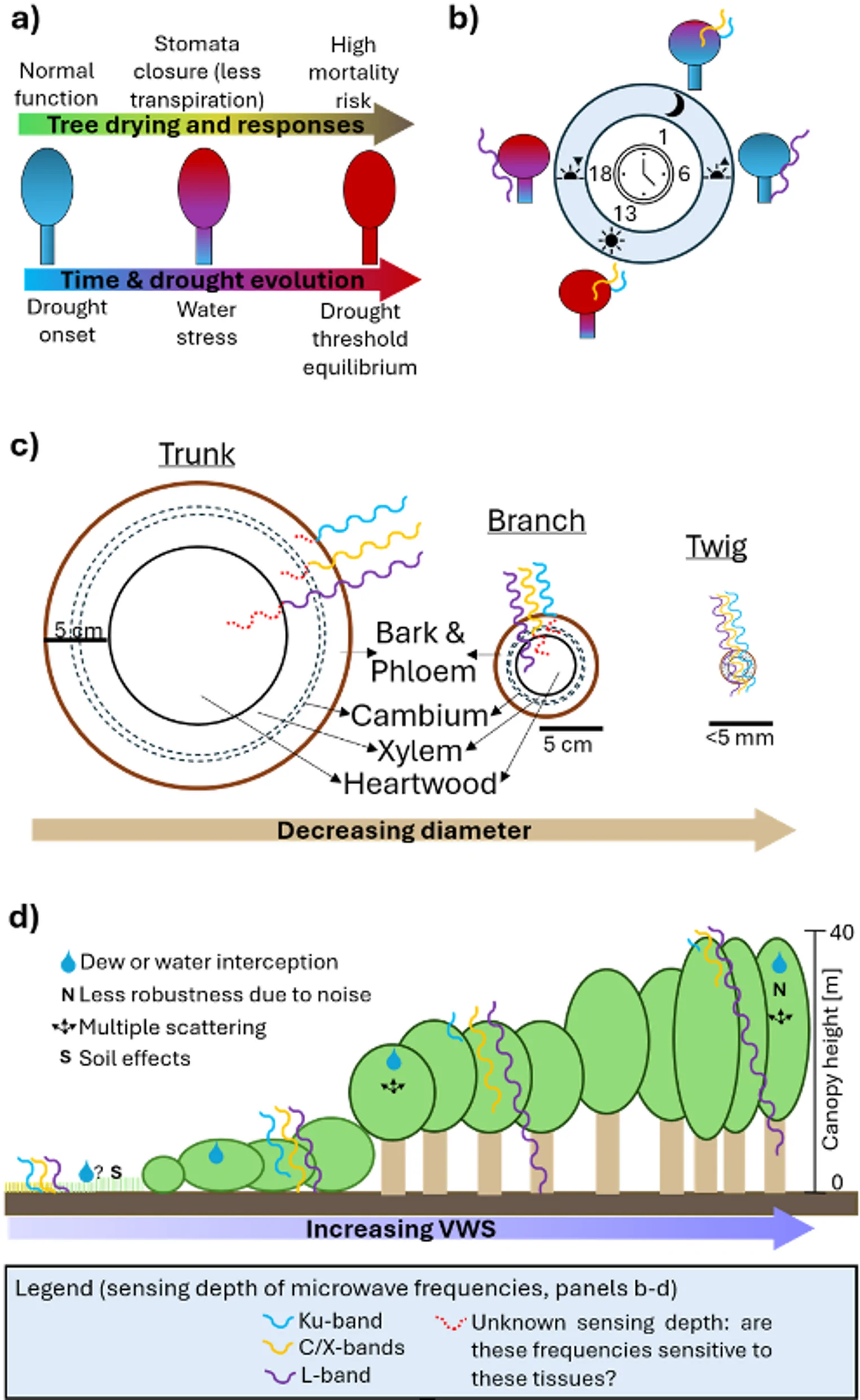
Conceptual representation of remote sensing of vegetation moisture from passive microwaves. **a** Drought evolution, which can be sensed by microwave satellites, progressively leads to increased water stress and finally to drought-mediated equilibrium due to large water depletion [68], which involves high mortality risk. **b** The data availability of different satellite microwave frequencies and their sensing depth through the canopy is represented in colored waves (see the bottom legend), together with the distribution of the water vertical profile in forests at the satellite time overpasses (in a 24-h clock, with sunrise, midday, sunset and night represented). Note the higher drought stress at midday, when L-band data is unavailable. At midday, static atmospheric conditions are assumed. **c** The drawing represents microwave interactions at different frequencies with decreasing diameters. This includes a trunk (left), a branch (center) and a twig or an herbaceous stem (right; note the mm scale). Microwave sensing depth across the thickness of forest tissues is known only in some cases (~ 5 cm for L-band according to [128]). It requires deeper research to determine which frequencies (and for which thicknesses) can sense the physiologically active fractions of vegetation stems (red dashed waves). **d** Microwave sensing depth for increasing biomass and vegetation water content, and the limitations linked to each vegetation type

Going beyond the estimation of water content in the spatiotemporal domain, it is paramount to consider the vertical distribution of water content and water potential in the canopy, which is responsible for driving the water flux through the soil-plant-atmosphere system. Before dawn, plants are typically near hydraulic equilibrium. This means that roots, xylem and leaves should reach similar water potential values due to overnight rehydration, when root uptake outpaces transpiration [126]. In contrast, the maximum water demand generally occurs in the middle of the day, when the maximum transpiration rate and water potential gradient between the soil and the leaves are reached (Fig. 5b). Under drought, the stomatal closure reduces transpiration, which progressively diminishes the water potential gradient until reaching a drought-induced equilibrium [68] and potentially leads to increased plant mortality and canopy die-back [127]. Hence, the absence of a water potential gradient during the day should by extension represent drought stress (Fig. 5a and [68]).

The use of VOD from multiple frequencies may provide insights into the relative magnitude of the gradient from the upper canopy (high frequencies) to the entire vertical profile (L-band) [24]. Under non-stressed conditions, we expect a big difference to emerge in the ratios of different VOD frequencies between predawn (minimal gradient) and midday (significant gradient). In contrast, we expect the difference between predawn and midday ratios to be close to zero under drought conditions. This is because a reduced daily gradient would indicate the onset of drought (Fig. 5a). One challenge of using the ratios of different VOD bands is that, ideally, the retrievals at each band should have been taken simultaneously. As depicted in Fig. 5b, satellite-based L-band measurements are available at dawn (SMOS/SMAP, ~ 6 a.m.), while C- to Ku-band acquisitions are available overnight (AMSR-E/2&3; ~1:30 a.m.). Interpreting those in the context of hydraulic equilibrium requires the assumption that vegetation has reached equilibrium with the soil by ~ 1:30 a.m [129]. Midday overpasses are available from some optical sensors (e.g., MODIS, ~ 1:30 p.m.) and from C-/X-/Ku-band data as well (AMSR-E/2&3; ~1:30 p.m.). Thus, comparing midday with night ratios between frequencies is only currently feasible with the high-frequency measurements of AMSR-E/2&3. As of 2029, the Copernicus Imaging Microwave Radiometer satellite mission (CIMR) is expected to provide pre-dawn multi-frequency overpasses, ensuring collocated measurements at L- to Ku-bands which should facilitate the analysis of hydraulic gradients in VOD data due to coinciding, multi-frequency measurements, allowing the reconstruction of the vertical profile across the vegetation [130].

In contrast, L-band measurements (entire vegetation profile) [54–56], are not currently available in the middle of the day (Fig. 5b), thus limiting the assessment of gradients in forest stems using currently available passive microwave satellites. The launch of sub-daily missions has been theoretically proposed in the form of a multi-satellite L-band synthetic aperture radar constellation [24, 131]. The future realization of this or other lower frequency sub-daily missions [24] can improve our capacity to monitor vegetation function and drought stress.

#### Sensitivity of Different Microwave Frequencies to Different Plant Tissues

Importantly, while the vertical sensing depth of microwaves through canopies is understood to vary with frequency [54–56], the depth of plant tissue that is represented by the signal is largely unknown (Fig. 5c and [68]; however, also see [132] and [133] for exemplary studies). Plant stems and branches comprise tissues arranged concentrically. First, there is a thin layer of bark and phloem on the outer surface (< 10 mm). The phloem transports carbohydrates from the leaves to the stem and roots. Second, the xylem includes sapwood conducting water upwards from roots to leaves. Finally, the heartwood (‘inactive’ xylem) is in the center of woody plants (Fig. 5c). Understanding the link between microwave remote sensing and tissue-specific conditions, combined with information on plant vertical water gradients, can bring invaluable capacity to assess vegetation conditions and functions in ecophysiology.

L-band measurements have been found to correlate with xylem water potential [43, 44], but little is known about whether this and other frequencies are also sensitive to processes in the phloem. Further research will have to be conducted to determine the relationship between the microwaves’ dielectric constant and attenuation, and the flux of water and sugars through the phloem. Such an analysis will need to account for the impact of solute concentration in dielectric models, which is only assumed as a constant and empirical value in [134], and ignored in other models (e.g. [135]). Note that the role of phloem and solute transportation may be essential in plant drought-induced mortality, which can be driven by xylem cavitation but also by carbon starvation [136]. Another gap in the current state-of-the-art is whether microwave emissions and backscatter are sensitive to the water stored in the heartwood. This is especially the case of branches or stems thicker than 5 cm, a depth to which L-band has been found sensitive [132] (Fig. 5c).

Similarly, it is likely that C-band VOD is sensitive to the water in the sapwood and phloem in branches, however, the full extent of its sensitivity through the branch thickness is not known and needs more research, while Ku-VOD may only be representative of fine twigs or thin branches (Fig. 5c). These different assumptions have direct physiological implications because, to relate an amount of water to the current physiological state of the vegetation, one needs to know which part of the system is sensed. As discussed above, this is a complex issue because the behavior of microwaves varies not only as a function of tissue and observation frequency but also depends on the viewing geometry (incidence and aspect) and polarization.

#### Need for Enhanced Dielectric and Retrieval Models

Likewise, different tissues and plant types may require the application of specific dielectric models to retrieve their water content from microwave remote sensing. However, we have found that this is not the case in the literature. For instance, the same dielectric model by Ulaby & El Rayes, based on laboratory permittivity measurements of free water and sucrose-based solutions [134], has been used to link the dielectric constant with vegetation moisture either in a wheat field [42] or in both the trunk’s xylem and the leaves of an oak forest [43]. Furthermore [35], used two models based on dielectric measurements of leaves [128, 135] in comparison with Ulaby & El Rayes [134] and applied those to different ecosystems and variations of leafy or woody tissues to retrieve LFMC. Although all three models showed a reasonable performance in estimating LFMC (*r* > 0.5 at X-band), they also showed significant differences in the relationship between vegetation moisture and dielectric permittivity (see the supplementary information of [35]). Thus, the application of dielectric models to retrieve water in vegetation seems to be arbitrary so far. Consequently, there is a need for improvement and implementation of tissue-specific dielectric models, especially with a focus on assessing the dielectric constant in woody tissues (e.g. [137]), and its relationship to the xylem and phloem conditions.

There are various limitations in monitoring water in vegetation with VOD that should be understood. First, VOD retrieval models consider only single scattering effects in forests, while multiple scattering is expected. Taking it into account significantly increases VOD values [138]. Second, in dense forests there is a lower retrieval robustness due to noise in single-angle radiometer instruments like SMAP. Solving this requires modifying the current regularization approaches in VOD retrievals [139]. Third, the surface canopy water from interception and dew may prevent VOD from providing information about plant water stress in tropical forests [140] (Fig. 5d). Finally, it should be further explored whether soil emissions and roughness are correctly accounted for in VOD retrieval algorithms, especially in short vegetation (Fig. 5d). All these may compromise the quality of vegetation water estimates and need further research to improve current retrieval models.

## A Road Towards Monitoring Vegetation Water Potential (ψ) from Remote Sensing

### Linking Water Content to Water Potential: From *In situ* Measurements to Remote Sensors

Water potential mechanistically relates to physiological processes such as stomatal conductance (influencing rates of photosynthesis [141]) and to hydraulic traits including conductivity thresholds (influencing drought induced mortality [136]). Therefore, Ψ is a central component of plant hydraulic monitoring using models [142], and it is needed to improve drought response predictions [143]. The relationship between θ (or RWC_phys_) and Ψ, known as the pressure-volume (PV) curve (Figs. 4 and S7), has been characterized for leaves of many species and in woody tissues of some species [67, 144]. In the region of the PV curve of minimal water stress, the PV relationship tends to be almost linear, after which there is an inflection point indicating a threshold towards water stress (i.e., the turgor loss point in leaves [67], or the cavitation threshold in the xylem [145, 146]; Figure S7).

While PV relationships are increasingly well understood in isolated plant tissues, less is known about how Ψ and θ or RWC_phys_ vary across tissues and spatiotemporal scales [68] and it is thus challenging to create an ecosystem-level PV curve. It has been common practice to measure leaf water potential at predawn and midday in field sites [127], but less common to measure leaf θ or stem/branch Ψ or θ. Moreover, the measurement of leaf Ψ tends to focus on sampling extreme values (e.g., upper sunlit leaves), rather than ecosystem average values [68]. Consequently, we have limited understanding of the relationship between θ and Ψ beyond tissue scales, and similarly for how Ψ varies across systems [70]. Recently, a new global network of *in situ* Ψ measurements (PSInet) has been published. It will facilitate the potential to calibrate, compare and even validate future remote sensing Ψ estimates [45].

Until now, most Ψ-related remote sensing research has focused on studying correlations between optical indices and plant Ψ, mainly at the local scale (Fig. 1). These studies usually assess crops (see ‘Supplementary references’). Concerning microwaves, the relationship between VOD and Ψ has been explored in a forest stand using a tower-based radiometer [44]. Also, the joint effects of Ψ, biomass, and LAI on the VOD signal have been characterized in [147]. However, only recent research has provided estimates of water potential based on a synergy between remote sensing retrievals of plant water content and models of plant water relations [43, 72]. In these cases, water potential was estimated with plot-scale radiometers in a wheat field [72] and in a forest stand [43] following four steps. First, the effect of the dielectric constant of vegetation (ε_veg_) on VOD was disentangled from the effect of temporal variations in biomass. Previous studies have done this by either considering only sufficiently short periods when biomass variations were low [43] or by using Sentinel-1 radar in a semi-physical model accounting for biomass variations [35]. Second, dielectric models were applied to estimate the gravimetric vegetation moisture (m_g_; Table 1) from ε_veg_ [35, 42]. Third, m_g_ was normalized to calculate RWC_RS_ [%] (instead of directly normalizing VOD as in Table 1). Fourth, Ψ was estimated from RWC_RS_ by using a sigmoidal function with empirically determined parameters defined in [148] which aims at approximating the behavior of PV curves. Other examples assume linear relationships between VOD, RWC and Ψ [44], which may exist within the normal physiological range of plant function, but cannot estimate Ψ responses beyond the turgor loss point [68] (Figure S7).

### Bridging the Fields of Ecophysiology and Remote Sensing to Retrieve Water Potential

Despite these recent efforts, further research is needed to close the gap between remote sensing and ecophysiology. First, we recall that RWC_RS_ is by no means equivalent to RWC_phys_ (Sect. "Monitoring the water status of vegetation"), which limits its applicability in pressure-volume relationships. This limitation can be overcome by using θ and VWS [68] (Sect. "Linking satellite and *in situ* water content information") as the basis to estimate Ψ (Figure S7), instead of using RWC.

A second issue is that the new remote sensing research pursuing Ψ estimates has approximated the concept of PV curves by using empirical sigmoidal or linear functions [43, 44, 72]. This can be enhanced by applying recently defined, more realistic ecosystem-scale PV curves (EPV curves) to upscale water relations to the plot level [24, 68, 69, 149]. Here we propose a theoretical framework applying EPV curves for a remote sensing-based estimation of Ψ in plots (e.g., GNSS-T sites of up to ~ 100 m in aerial diameter). We also suggest a theoretical approach to Ψ estimates at the scale of passive microwave pixels (0.25º). For these purposes the required data are listed in Table 4, and three steps should be followed:

1. Obtain data on hydraulic traits: First, hydraulic traits have to be measured at plot or site level or obtained from literature or auxiliary information for the species present in that site ([67, 68, 122], and Table 4). These traits are the basis for building tissue- or tree-scale PV relationships (Figure S7). At the pixel level, the same literature and auxiliary sources could be used. In addition, knowledge on which species are present inside the pixel and their spatial distribution can be obtained from national forest inventories (NFIs).
2. Define the EPV curves: Second, it is necessary to mathematically define the function linking volume and pressure. To do so at the plot level one can refer to [68], where the relationship between the plot-scale VWS and Ψ is established based on the θ of trees and uses the wood volume ($\:{V}_{wood}$, Eq. (7)) and the LAI (Eq. (8)) as upscaling variables:

7 $${VWS\left({\Psi}\right)}_{wood}={\rho}_{water}.\sum_{tree=1}^{tree=n}{V}_{wood}.{\theta\left({\Psi}\right)}_{wood}/{A}_{plot}$$

8 $${VWS\left({\Psi}\right)}_{leaf}=LAI.{\theta\left({\Psi}\right)}_{leaf}$$

where $\:VWS\left({\Psi\:}\right)$ is the EPV plot-scale function and $\:\theta\:\left({\Psi\:}\right)$ is the PV curve of the wood or leaves (Figure S7). We note that, in non-woody ecosystems, only Eq. (8) should be applied.

At the remote sensing pixel (0.25º) scale, the upscaling variables wood volume (i.e., based on biomass and wood density) or LAI can be derived from datasets in Table 4. The fact that (i) these datasets and the species presence maps are available at < 1 km resolution and (ii) the traits information is available at the species level, allows us to: (i) approximate with a certain detail the spatial distribution of traits within a 0.25º pixel, and (ii) build weighted means of traits at the 0.25º scale using LAI, volume and species spatial information. As a result, pixel-scale EPV curves could be approximated also based on (7) and (8) considering the pixel area instead of the plot area. Although this approach is purely theoretical, data on species abundances have been recently used to create pixel-weighted means of plant traits [121], confirming the potential of this concept. While this approach is species-based, trends in intraspecific variation by region or climate should also be explored in the future [89].

**Table 4:** Data needed to: (i) estimate θ as in Eq. (3) to (6), (ii) build PV curves, and (iii) build plot- and pixel-scale EPV curves that can be applied to estimate Ψ from remote sensing (Eqs. (7), (8))

Data		Plot scale approach	Pixel scale analogy
θ estimation (Eqs. 3 to 6)	LFMC	Stems: likely from tower radiometry [43] or GNSS-T (but not done yet). Leaves: e.g., tower spectrometers	Stems: L-band-VOD-derived LFMC [35] Leaves: products in Table 2
	VWS	N/A^1^	Stems: L-band VOD-derived VWS [25] Leaves: [27] or [30]
	ρ_wood_	Stems: *In situ* data	Global map based on machine learning and ~ 30,000 *in situ* measurements [117]
	B_wood_	Stems: *In situ* data	Global biomass maps (e.g., [118])
	LAI	*In situ* data or TLS	Global LAI maps [119]
	SLA	*In situ* data	Global database of leaf traits based on remote sensing [120, 121]
Traits for PV curves (Figure S7)	Ψ_thr_	*In situ* data, literature (e.g., [67]), and phylogeny [122]	Literature (e.g., [67]) and phylogeny [122]
	θ(Ψ_max_)		
	Ψ_max_	Canopy height (see Table S1 in [68])	Canopy height maps [123, 124]
EPV curves	Number or proportions of species	Field knowledge on the number of trees for each species	Pixel-weighted means of species proportions based on NFIs
	Stem volume	*In situ* data or TLS	Biomass /$\:{\rho\:}_{wood}$ [117, 118]
	Leaf area	*In situ* data or TLS	LAI · (pixel size)
	Area	Plot area	Pixel grid size

^1^Using Eqs. (1) and (2) would introduce redundancy since θ is needed to estimate VWS and vice versa

3. Plot- or pixel-scale Ψ estimates: Finally, the EPV curves defined in the previous step can be used to derive plot- or pixel-scale Ψ estimates. This requires using remote sensing plot- (GNSS-T) or pixel-scale (satellite-based) estimates of VWS or θ (Sect. "Linking satellite and *in situ* water content information" and Fig. 4).

Importantly, note that scaling hydraulic traits per ground area is challenging, with uncertainty associated with each data source (Table 4). This represents the forefront of ecophysiological field research. However, it is conceptually similar to the big-leaf models used for decades to estimate fluxes in soil-plant-atmosphere models [150], but with explicit representation of the vegetation water storage component.

## Conclusions and Recommendations

In this paper, we review the literature available on remote sensing of water in vegetation across scales and variables, including LFMC, water content (θ, VWS, RWC) and water potential (Ψ). We find that, independently of the variable studied, local-scale research largely dominates the literature, underlining that knowledge of large-scale properties is still limited (Fig. 1a). We have identified several research gaps and highlighted possible methods to improve large-scale monitoring of water in vegetation, especially from satellite technologies and their synergy with emerging networks of *in situ* data [19, 41, 45]. These networks have not yet been used to their full potential to link remote sensing with ground-based research and to validate or intercompare remote sensing products for the assessment of vegetation water status (Fig. 1b). These gaps can be summarized in two research questions: (i) Which strategies are needed to achieve and/or enhance large-scale products of water in vegetation? (ii) How can ground-based data be better integrated to establish a stronger link with remote sensing estimates? Table 5 addresses these questions by providing a list of recommendations derived from this paper.

**Table 5:** Recommendations derived from this paper for each variable. Symbols are consistent with those in Fig. 1 and refer each one to a research question: Which strategies are needed to achieve and/or enhance large-scale products of water in vegetation? How can ground-based data be better integrated to establish a stronger link with remote sensing estimates?

Var.	Symbol	Recommendation
LFMC	◼	Different technīques of LFMC satellite products based on machine learning, RTM and VOD-LAI models capture well time-dynamics in the western USA and in the Mediterranean (Table 2). In the western USA, consider using the VOD-based semi-physical algorithm by [35] or the optics-radar synergy in [37] to capture absolute LFMC values, sincethey have the lowest RMSE and bias in the region. In Australia, onecan apply [50] and [51] which are the best performing products.
	▲	For validation purposes of LFMC satellite products, consider thehomogeneity of species as well as if they are representative of the dominant vegetation type in the pixel. Also, consider the variability in time of the *in situ* data. We hypothesize that these filters can be applicable also to future satellite vegetation water products.
VWS	◼	Improve satellite VWS estimates by (i) enhancing estimates of the b-parameter, (ii) applying different VOD frequencies to produce maps of VWS for different canopy fractions, and (iii) further exploring the synergy between signals of opportunity (e.g., GNSS) with Al.
	▲	To validate satellite VWS maps, use VWSstem and VWSleaf plot-scaleestimates upscaled from *in situ* 0 measurements and TLS data ofwood volume and leaf area (see Eqs. (1) and (2)), or develop plot-scale VWS estimates based on GNSS-T VOD data [74]. Vegetation in the benchmark plots should be representative of the vegetation in the pixel.
	▲	Add field SLA and LAI data to *in situ* LFMC databases. It can be applied to derive Oleaf data, serving as a linking variable between the research fields of remote sensing, fire risk assessment and plant ecophysiology.
θ	◼	Develop 0 datasets based on satellite VWS or LFMC, and on biomass, wood density and/or LAI and SLA maps (Eqs. 3 to 6 and Table 4).
	◼	Plan satellite L-band missions with sub-daily overpasses (e.g., SLAINTE), to complete our capacity to assess vertical gradients ofwater in forests.
Ψ	▲	Conduct laboratory and field experiments to determine the sensitivity of microwave frequencies to the water content in different plant tissues, and for different thicknesses of branches and trunks. Develop more specific dielectric models tailored to, for instance, specific plant tissues or to species composition in study pixels.
	◼	Further explore the theoretical framework proposed to develop plot-scale and satellite remote sensing estimates of Y based on EPV curves, weighted means of plant traits and remote sensing estimates of water content (Section 4 and Table 4). Validate the estimates with *in situ* PSInet data where available [45].

Overall, our review highlights the need to further combine *in situ* data and satellite remote sensing retrievals. This joint improvement includes (i) accounting for more sophisticated filters of *in situ* data for validation strategies of satellite products, (ii) deriving water content information at plot and pixel scales, (iii) validating satellite VWS estimates using upscaled water content information from *in situ* and GNSS-T-derived VWS [74], and (iv) reconciling the remote sensing approach to estimate RWC_RS_ with ground-based ecophysiological variables (RWC_phys_). In addition, remote sensing of water in vegetation needs to account for the variability of plant water status across the vertical gradients of the plant. The gradients change throughout the day and in drought conditions. To limit the effect of vertical profiles of water status on variations in observations, multiple frequencies to estimate VOD need to be exploited, emphasizing the need for future passive microwave missions providing data at sub-daily timescales to capture predawn and midday plant water status in forests. The capacity of microwave frequencies to sense different tissues depending on their sensing depth is also discussed. Future research has to be carried out to determine which microwave frequencies are best suited to sense the xylem and the phloem, as a new step to further understand forest responses to drought stress (Sect. "Relationship of microwave sensing depth to the water status from different plant heights and tissues").

Finally, a new theoretical framework is proposed to estimate Ψ from remote sensing at plot (e.g., from GNSS-T VOD) and satellite scales (from satellite VOD). Remote sensing-based Ψ estimates will require, as a first step, to monitor VWS (Sect. "Linking satellite and *in situ* water content information"). Afterwards, novel approximations like the use of plot- or pixel-scale EPV curves should be applied to derive Ψ from these VWS estimates. When conducted at the satellite scale, this approach requires merging new global maps of wood density [117] and biomass [118] with forest inventories and ground-based ecophysiological information on plant hydraulic traits to upscale pressure-volume relationships (Table 4).

The proposed frameworks and recommendations can improve understanding of LFMC products for fire risk assessment and open new perspectives on plant ecophysiology, extending it from current point-scale measurements to potential large-scale estimates of key variables regularly in time and space. This can represent a significant step towards determining forest mortality thresholds, enhancing our understanding of plant water relations and including information on plant water status in Earth system mode .

## Supplementary Information

Supplementary Material 1.

## Data Availability

The compared LFMC products are publicly available following data availability statements in references of Table 2 and/or upon request to the authors.

## Key References

Papers of particular interest, published recently, are highlighted here:

•• Of major importance

- [19] Yebra M, Scortechini G, Adeline K, Aktepe N, Almoustafa T, Bar-Massada A, et al. Globe-LFMC 2.0, an enhanced and updated dataset for live fuel moisture content research. Sci Data. 2024;11(1):332. This paper details the updated first-time global dataset of *in situ* LFMC samples. The dataset has been essential for calibration of LFMC estimates and is used here for the comparison of LFMC datasets in Section 2.
- [45] Restrepo-Acevedo AM, Guo JS, Kannenberg SA, Benson MC, Beverly D, Diaz R, et al. PSInet: A new global water potential network. Tree Physiol. 2024;44(10):tpae110. This is the first global dataset of *in situ* vegetation water potential measurements. It should serve as the key calibration and validation tool for potential remote-sensing-based estimates of Ψ.
- [68] Binks O, Meir P, Konings AG, Cernusak L, Christoffersen BO, Anderegg WR, et al. A theoretical framework to quantify ecosystem pressure–volume relationships. Glob Change Biol. 2024;30(11):e17567. This paper proposes and applies a framework to develop EPV curves at plot scales, which is essential for future large-scale research of plant water relations and for potential estimations of water content and water potential from remote sensing sources.

• Of importance

- [35] Chaparro D, Jagdhuber T, Piles M, Jonard F, Fluhrer A, Vall-Llossera M, et al. Vegetation moisture estimation in the Western United States using radiometer–radar–lidar synergy. Remote Sens Environ. 2024;303:113993. This paper presents the first LFMC estimates for different microwave frequencies (i.e., for different canopy layers). The study is conducted in the western USA. Its main innovation is the fact that it isolates the water component of VOD from the biomass/structure component of this signal.
- [36] Forkel M, Schmidt L, Zotta RM, Dorigo W, Yebra M. Estimating leaf moisture content at global scale from passive microwave satellite observations of vegetation optical depth. Hydrol Earth Syst Sci. 2023;27(1):39–68. This paper presents the first microwave-based global LFMC estimates. It implicitly separates water and biomass effects in the VOD signal by combining VOD data with LAI information.
- [37] Rao K, Williams AP, Flefil JF, Konings AG. SAR-enhanced mapping of live fuel moisture content. Remote Sens Environ. 2020;245:111797. This work presents an innovative synergy between radar and optical satellite remote sensing to provide enhanced high-resolution LFMC estimates in the western USA.
- [43] Jagdhuber T, Schmidt AS, Fluhrer A, Chaparro D, Jonard F, Piles M, et al. Estimation of forest water potential from ground-based L-band radiometry. IEEE J Sel Top Appl Earth Obs Remote Sens. 2025;18:5509–22. This paper estimates, for the first time, the water potential of a forest stand based on local-scale microwave remote sensing.
- [74] Feldman AF. Emerging methods to validate remotely sensed vegetation water content. Geophys Res Lett. 2024;51(14):e2024GL110505. This paper proposes different ways (including *in situ* and GNSS-T data) to validate satellite VOD products, bridging the gap between *in situ*, plot and satellite scales for VOD accuracy assessment.
