# Supplementary material for: Monitoring Water in Vegetation: Mind the Gap Between Satellite Remote Sensing and Field Scale Approaches
Source: Curr For Rep. 2026 Nov 14;12(1):34. doi: 10.1007/s40725-026-00290-y (PMC13769691; doi:10.1007/s40725-026-00290-y)
Supplement: Supplementary file 1 [file 40725_2026_290_MOESM1_ESM.docx]

**Supplementary material**

**Supplementary Information 1: description of the methodology used to create Figure 1**

The literature search for Figure 1 has been conducted in Google Scholar for the period January 2014 - October 2024, both included (~11 years). The search has considered local, regional, continental and global scales. We have searched for two groups of variables, corresponding to the two columns in Figure 1: water content and water potential. Tables S1 and S2 detail the search terms used, which refer to the variables within each group in Table 1. First, for each variable within each group, the first *n* papers from the “ordered by relevance” list of the search engine have been checked. The number *n* is proportional to the number of search outputs for that variable (Tables S1 and S2). The total number of papers checked for each group has been limited to 200 papers. Second, from the *n* checked papers, *m* relevant papers for each variable have been chosen and reviewed if they meet the following criteria:

1. The work is a research paper, a PhD thesis, or a conference abstract long enough to obtain detailed information (e.g., short, ~500-word (one-page) abstracts were excluded). Review papers are excluded since they can add redundancy with other reviewed papers.
2. The research describes remote sensing-based products or retrieval approaches, details the use of radiative transfer models to derive the variable under study, analyzes correlations among the studied variable and remote sensing information, or applies remote sensing-derived products of the variable under study.
3. The remote sensing sensor is carried on a satellite, an airplane, a UAV, or a tower, or a similar mounted sensor. We exclude handheld sensors and laboratory spectrometers.
4. The study area is clearly defined.

For instance, in the group “water content”, *n* = 62 out of the 200 papers investigated (31.2%) correspond to the variable “vegetation water content”, since 7580 out of 24328 outputs in this category (31%) are related to this variable. A total of *m* = 15 out of the 62 papers meet the criteria and are selected as relevant papers and therefore reviewed (Tables S1).

For each group, the *m* relevant (reviewed) papers have been qualitatively classified in four different spatial scales:

- Local: this includes, for instance, tower-based sensors or field research, as well as local areas such as one or a few municipalities, or a natural park. Note that studies including many local sites distributed through large regions (even continents or the globe) are also considered as local scale research, if no wall-to-wall maps of the studied variable are shown in the results (i.e., the study is only at the point scale even if the sites are widely distributed).
- Regional: examples are provinces, countries that do not span sub-continental scales (e.g., Portugal or Italy), or large geographic regions such as the Iberian Peninsula or the western United States.
- Continental: includes continents but also very large areas or countries. Examples are Africa, Europe, the contiguous United States (CONUS), China or Australia.
- Global: includes global or quasi-global datasets or research.

Therefore, Figure 1 is built as follows. First, the *m* reviewed papers are classified for each combination of spatial scale and group. Second, a research gap is defined when ten or fewer papers are found in a scale-group combination. Third, for each gap, and based on the qualitative review of the papers, challenges are highlighted and used to build the two main research questions (Figure 1). We acknowledge that this approach to detect challenges is qualitative and cannot be fully objective.

A list of the references for all the relevant (reviewed) papers is found at the end of this supplementary document. Note that the search method applied is limited to the search words employed. Still, we have checked that these omissions are not relevant to the result in Figure 1, in which the search conducted is representative enough of the current literature to properly detect gaps through variables and scales.

**Supplementary Information 2: details on the methodology used to compare LFMC filters and products in Section 2.3**

The comparison of filters and products undertaken in Section 2.3 has two main steps.

1. In the first step, our research explores the effect of filters in Table 3 on *r*, RMSE and bias statistics derived from the comparison between *in situ* and remote sensing datasets. We note that this first step excludes Australia from the analysis since the number of available sites there is small and is excessively reduced when filters are applied. Also, the LFMC datasets covering the entire CONUS are not included since they are not publicly available [79] or do not match in time with other datasets [78] (Table 2). We use only sites where the number of *in situ* LFMC samples is larger than ten, and these *in situ* samples are temporally coincident with data from all the LFMC products under study in each region. Figure S1 illustrates which sites are used. All LFMC products have been aggregated to the coarsest grid among the products studied (0.25º) and limited to the same time periods (Table 2). In [74], [57•] and [85] time interpolation was needed since temporal resolutions of these products range between 7 and 15 days, compared to almost daily data availability in the other products.

The different filters are explained in Table 3. Here, we provide further details on the Std_site_ filter. This filter is motivated by the fact that interpreting the Pearson’s correlation coefficient (r) cannot be done without considering the time-variability of the *in situ* time series. This is because the correlation between time series with low time variability is dominated by observational or system-rooted noise and does not provide a meaningful basis for comparison [29•, 57•]. To address this issue, the coefficient of variation of the *in situ* time series has been studied as a metric to decide which sites offer a better comparability (i.e., a wide enough variability in time to avoid the time series being dominated by noise) [57•]. In [29•] the metric is adapted by using the standard deviation (Std_site_). Thus, in Table 3, we have added Std_site_ as a filter to be considered in validation and comparison activities.

2. In the second step we use the best filtering strategy (see Section 2.3) to compare LFMC products with one another. In this step, only sites with ten or more *in situ* measurements are used. However, we make an exception for Australia. We include it in the product comparison analysis but, due to the small number of sites and samples available, we do not apply quality filters and reduce the minimum number of samples per site to n=7.

**Supplementary tables**

**Table S1.** Search approach for the group water content.

**Table S2.** Search approach for the group water potential.

**Table S3.** Number of *in situ* LFMC samples in each region. Left column: all sites available; middle column: sites with ≥10 samples (≥7 in Australia) used in the study of filters (Figure 2); Right column: sites remaining after applying the screening filters (i.e., the more representative sites), which are used in the comparison analyses (Figures 3 and S2 to S6), except for Australia. Bold font: the number of study sites used in each region for Figures 3 and S2 to S6.

| Region | All sites | N≥10 (≥7 in Australia) | Filters applied |
| --- | --- | --- | --- |
| Western USA | 750 | 332 | **78** |
| Mediterranean | 208 | 81 | **10** |
| Australia | 43 | **19** | Not applied |

**Supplementary figures**

**Figure S1.** Sites of field scale LFMC measurements from Globe-LFMC v2 [19••] used in the study of filters and product intercomparison (red) or only in the study of filters (black). Grey dots represent the excluded sites. Regions: (a) western United States, (b) Australia, (c) the Mediterranean basin.

**Figure S2.** Mediterranean basin. Effect of progressively stricter site-screening criteria (as in Table 3) on the agreement (median correlation) between satellite-derived LFMC products and *in situ* LFMC measurements. The satellite LFMC products are represented by color lines. The legend provides the products’ references. The subfigures show: (a) the maximum spatial coefficient of variation of NDVI at a site (CV_NDVI,Max_), (b) the land cover ‘distance’ between 1-km and 0.25º pixels (D_LC_), (c) the filter removing LFMC sites with more than one species sampled (Sp1), (d) the percentage of sampled species which are of the same vegetation type as the dominant in the pixel (Sp2), (e) land cover homogeneity as the percentage of dominant land cover in the pixel (LC_homog_) and (f) standard deviation of *in situ* LFMC (Std_site_).

**Figure S3.** Western USA. Effect of progressively stricter site-screening criteria (as in Table 3) on the agreement (median RMSE) between satellite-derived LFMC products and *in situ* LFMC measurements. The satellite LFMC products are represented by color lines. The legend provides the products’ references. The subfigures show: (a) the maximum spatial coefficient of variation of NDVI at a site (CV_NDVI,Max_), (b) the land cover ‘distance’ between 1-km and 0.25º pixels (D_LC_), (c) the filter removing LFMC sites with more than one species sampled (Sp1), (d) the percentage of sampled species which are of the same vegetation type as the dominant in the pixel (Sp2), (e) land cover homogeneity as the percentage of dominant land cover in the pixel (LC_homog_) and (f) standard deviation of *in situ* LFMC (Std_site_).

**Figure S4.** Mediterranean basin. Effect of progressively stricter site-screening criteria (as in Table 3) on the agreement (median RMSE) between satellite-derived LFMC products and *in situ* LFMC measurements. The satellite LFMC products are represented by color lines. The legend provides the products’ references. The subfigures show: (a) the maximum spatial coefficient of variation of NDVI at a site (CV_NDVI,Max_), (b) the land cover ‘distance’ between 1-km and 0.25º pixels (D_LC_), (c) the filter removing LFMC sites with more than one species sampled (Sp1), (d) the percentage of sampled species which are of the same vegetation type as the dominant in the pixel (Sp2), (e) land cover homogeneity as the percentage of dominant land cover in the pixel (LC_homog_) and (f) standard deviation of *in situ* LFMC (Std_site_).

**Figure S5.** Western USA. Effect of progressively stricter site-screening criteria (as in Table 3) on the agreement (median bias) between satellite-derived LFMC products and *in situ* LFMC measurements. The satellite LFMC products are represented by color lines. The legend provides the products’ references. The subfigures show: (a) the maximum spatial coefficient of variation of NDVI at a site (CV_NDVI,Max_), (b) the land cover ‘distance’ between 1-km and 0.25º pixels (D_LC_), (c) the filter removing LFMC sites with more than one species sampled (Sp1), (d) the percentage of sampled species which are of the same vegetation type as the dominant in the pixel (Sp2), (e) land cover homogeneity as the percentage of dominant land cover in the pixel (LC_homog_) and (f) standard deviation of *in situ* LFMC (Std_site_).

**Figure S6.** Mediterranean basin. Effect of progressively stricter site-screening criteria (as in Table 3) on the agreement (median bias) between satellite-derived LFMC products and *in situ* LFMC measurements. The satellite LFMC products are represented by color lines. The legend provides the products’ references. The subfigures show: (a) the maximum spatial coefficient of variation of NDVI at a site (CV_NDVI,Max_), (b) the land cover ‘distance’ between 1-km and 0.25º pixels (D_LC_), (c) the filter removing LFMC sites with more than one species sampled (Sp1), (d) the percentage of sampled species which are of the same vegetation type as the dominant in the pixel (Sp2), (e) land cover homogeneity as the percentage of dominant land cover in the pixel (LC_homog_) and (f) standard deviation of *in situ* LFMC (Std_site_).

**Figure S7.** Adapted from [64••]. A PV curve (blue) is built based on three tree hydraulic traits (red): the maximum water potential (Ψ_max_), the volumetric water content at the maximum water potential (θ(Ψ_max_)), and the threshold of water potential beyond which the shape of the relationship changes and water is lost faster than in normal conditions (Ψ_TLP_; where TLP refers to the turgor loss point in leaves). Classically in plant ecophysiology the water content in PV curves is expressed as RWC_phys_ instead of using θ but, as highlighted in Section 4.2, there is some limitation on how RWC can be estimated from remote sensing signals (RWC_RS_). The alternative of using θ respects the expected PV relationship and its monotonic variation and is also reported in literature [64••].
**Supplementary references**

References for relevant (reviewed) papers in Figure 1:

[G] Global; [C] Continental; [R] Regional; [L] Local.

*Water content*

[G] Oveisgharan, S., Haddad, Z., Turk, J., Rodriguez, E., & Li, L. (2018). Soil moisture and vegetation water content retrieval using QuikSCAT data. Remote Sensing, 10(4), 636.

[G] Chen, F., Liu, L., Guo, F., & Huang, L. (2024). A new vegetation observable derived from spaceborne GNSS-R and its application to vegetation water content retrieval. Remote Sensing, 16(5), 931.

[G] García-Haro, F. J., Campos-Taberner, M., Moreno, Á., Tagesson, H. T., Camacho, F., Martínez, B., ... & Gilabert, M. A. (2020). A global canopy water content product from AVHRR/Metop. ISPRS Journal of Photogrammetry and Remote Sensing, 162, 77-93.

[G] Quan, X., Yebra, M., Riaño, D., He, B., Lai, G., & Liu, X. (2021). Global fuel moisture content mapping from MODIS. International Journal of Applied Earth Observation and Geoinformation, 101, 102354.

[G] Fink, A., Jagdhuber, T., Piles, M., Grant, J., Baur, M., Link, M., & Entekhabi, D. (2018, July). Estimating gravimetric moisture of vegetation using an attenuation-based multi-sensor approach. In IGARSS 2018, IEEE International Geoscience and Remote Sensing Symposium (pp. 353-356). IEEE.

[C] Santi, E., Paloscia, S., Pampaloni, P., Pettinato, S., Nomaki, T., Seki, M., ... & Maeda, T. (2017). Vegetation water content retrieval by means of multifrequency microwave acquisitions from AMSR2. IEEE Journal of Selected Topics in Applied Earth Observations and Remote Sensing, 10(9), 3861-3873.

[C] Ebtehaj, A., & Bras, R. L. (2019). A physically constrained inversion for high-resolution passive microwave retrieval of soil moisture and vegetation water content in L-band. Remote Sensing of Environment, 233, 111346.

[C] Liu, L., Li, S., Yang, W., Wang, X., Luo, X., Ran, P., & Zhang, H. (2023). Forest canopy water content monitoring using radiative transfer models and machine learning. Forests, 14(7), 1418.

[C] Yebra, M., Quan, X., Riaño, D., Larraondo, P. R., van Dijk, A. I., & Cary, G. J. (2018). A fuel moisture content and flammability monitoring methodology for continental Australia based on optical remote sensing. Remote Sensing of Environment, 212, 260-272.

[C] Zhu, L., Webb, G. I., Yebra, M., Scortechini, G., Miller, L., & Petitjean, F. (2021). Live fuel moisture content estimation from MODIS: A deep learning approach. ISPRS Journal of Photogrammetry and Remote Sensing, 179, 81-91.

[C] Yebra, M., Quan, X., Riaño, D., Larraondo, P. R., Van Dijk, A. I., & Cary, G. J. (2018, July). Mapping live fuel moisture content and flammability for continental Australia using optical remote sensing. In IGARSS 2018, IEEE International Geoscience and Remote Sensing Symposium (pp. 5903-5906). IEEE.

[R] Wang, H., Wigneron, J. P., Ciais, P., Yao, Y., Fan, L., Liu, X., ... & Li, S. (2023). Seasonal variations in vegetation water content retrieved from microwave remote sensing over Amazon intact forests. Remote Sensing of Environment, 285, 113409.

[R] Zhang, Y., Bu, J., Zuo, X., Yu, K., Wang, Q., & Huang, W. (2024). Vegetation water content retrieval from spaceborne GNSS-R and multi-source remote sensing data using ensemble machine learning methods. Remote Sensing, 16(15), 2793.

[R] Liu, R., Wen, J., Wang, X., Wang, Z., Li, Z., Xie, Y., ... & Li, D. (2019). Derivation of vegetation optical depth and water content in the source region of the Yellow River using the FY-3B microwave data. Remote Sensing, 11(13), 1536.

[R] Chakroun, H., Mouillot, F., & Hamdi, A. (2015). Regional equivalent water thickness modeling from remote sensing across a tree cover/LAI gradient in Mediterranean forests of Northern Tunisia. Remote Sensing, 7(2), 1937-1961.

[R] Gao, Z., Wang, Q., Cao, X., & Gao, W. (2014). The responses of vegetation water content (EWT) and assessment of drought monitoring along a coastal region using remote sensing. GIScience & Remote Sensing, 51(1), 1-16.

[R] Lyons, D. S., Dobrowski, S. Z., Holden, Z. A., Maneta, M. P., & Sala, A. (2021). Soil moisture variation drives canopy water content dynamics across the western US. Remote
Sensing of Environment, 253, 112233.

[R] Fan, L., Wigneron, J. P., Xiao, Q., Al-Yaari, A., Wen, J., Martin-StPaul, N., ... & Kerr, Y. H. (2018). Evaluation of microwave remote sensing for monitoring live fuel moisture content in the Mediterranean region. Remote Sensing of Environment, 205, 210-223.

[R] García, M., Riaño, D., Yebra, M., Salas, J., Cardil, A., Monedero, S., ... & Ustin, S. (2020). A live fuel moisture content product from Landsat TM satellite time series for implementation in fire behavior models. Remote Sensing, 12(11), 1714.

[R] Wang, W., & Quan, X. (2023). Estimation of live fuel moisture content from multiple sources of remotely sensed data. IEEE Geoscience and Remote Sensing Letters, 20, 3002405.

[R] Rao, K., Williams, A. P., Flefil, J. F., & Konings, A. G. (2020). SAR-enhanced mapping of live fuel moisture content. Remote Sensing of Environment, 245, 111797.

[R] Cunill Camprubi, A., González-Moreno, P., & Resco de Dios, V. (2022). Live fuel moisture content mapping in the Mediterranean Basin using random forests and combining MODIS spectral and thermal data. Remote Sensing, 14(13), 3162.

[R] Xie, J., Qi, T., Hu, W., Huang, H., Chen, B., & Zhang, J. (2022). Retrieval of live fuel moisture content based on multi-source remote sensing data and ensemble deep learning model. Remote Sensing, 14(17), 4378.

[R] Argañaraz, J. P., Landi, M. A., Bravo, S. J., Gavier-Pizarro, G. I., Scavuzzo, C. M., & Bellis, L. M. (2016). Estimation of live fuel moisture content from MODIS images for fire danger assessment in Southern Gran Chaco. IEEE Journal of Selected Topics in Applied Earth Observations and Remote Sensing, 9(12), 5339-5349.

[R] Chaparro, D., Jagdhuber, T., Piles, M., Jonard, F., Fluhrer, A., Vall-llossera, M., ... & Entekhabi, D. (2024). Vegetation moisture estimation in the Western United States using radiometer-radar-lidar synergy. Remote Sensing of Environment, 303, 113993.

[L] Huang, Y., Walker, J. P., Gao, Y., Wu, X., & Monerris, A. (2015). Estimation of vegetation water content from the radar vegetation index at L-band. IEEE Transactions on Geoscience and Remote Sensing, 54(2), 981-989.

[L] Cosh, M. H., White, W. A., Colliander, A., Jackson, T. J., Prueger, J. H., Hornbuckle, B. K., ... & Bullock, P. (2019). Estimating vegetation water content during the soil moisture active passive validation experiment 2016. Journal of Applied Remote Sensing, 13(1), 014516.

[L] Small, E. E., Larson, K. M., & Smith, W. K. (2014). Normalized microwave reflection index: Validation of vegetation water content estimates from Montana grasslands. IEEE Journal of Selected Topics in Applied Earth Observations and Remote Sensing, 7(5), 1512-1521.

[L] Srivastava, P. K., O'Neill, P., Cosh, M., Lang, R., & Joseph, A. (2015, July). Evaluation of radar vegetation indices for vegetation water content estimation using data from a ground-based SMAP simulator. In IGARSS 2015, IEEE International Geoscience and Remote Sensing Symposium (pp. 1296-1299). IEEE.

[L] Kim, S. B., Huang, H., Liao, T. H., & Colliander, A. (2018). Estimating vegetation water content and soil surface roughness using physical models of L-band radar scattering for soil moisture retrieval. Remote Sensing, 10(4), 556.

[L] Chen, S., Chen, Y., Chen, J., Zhang, Z., Fu, Q., Bian, J., ... & Ma, Y. (2020). Retrieval of cotton plant water content by UAV-based vegetation supply water index (VSWI). International Journal of Remote Sensing, 41(11), 4389-4407.

[L] Sawada, Y., Tsutsui, H., Koike, T., Rasmy, M., Seto, R., & Fujii, H. (2015). A field verification of an algorithm for retrieving vegetation water content from passive microwave observations. IEEE Transactions on Geoscience and Remote Sensing, 54(4), 2082-2095.

[L] Wan, W., Larson, K. M., Small, E. E., Chew, C. C., & Braun, J. J. (2015). Using geodetic GPS receivers to measure vegetation water content. GPS Solutions, 19, 237-248.

[L] Zhu, X., Wang, T., Skidmore, A. K., Darvishzadeh, R., Niemann, K. O., & Liu, J. (2017). Canopy leaf water content estimated using terrestrial LiDAR. Agricultural and Forest Meteorology, 232, 152-162.

[L] Zhu, X., Skidmore, A. K., Darvishzadeh, R., & Wang, T. (2019). Estimation of forest leaf water content through inversion of a radiative transfer model from LiDAR and hyperspectral data. International Journal of Applied Earth Observation and Geoinformation, 74, 120-129.

[L] Hunt Jr, E. R., Daughtry, C. S., & Li, L. (2016). Feasibility of estimating leaf water content using spectral indices from WorldView-3’s near-infrared and shortwave infrared bands. International Journal of Remote Sensing, 37(2), 388-402.

[L] Miraglio, T., Huesca, M., Gastellu-Etchegorry, J. P., Schaaf, C., Adeline, K. R., Ustin, S. L., & Briottet, X. (2021). Impact of modeling abstractions when estimating leaf mass per area and equivalent water thickness over sparse forests using a hybrid method. Remote Sensing, 13(16), 3235.

[L] Traore, A., Ata-Ul-Karim, S. T., Duan, A., Soothar, M. K., Traore, S., & Zhao, B. (2021). Predicting equivalent water thickness in wheat using UAV mounted multispectral sensor through deep learning techniques. Remote Sensing, 13(21), 4476.

[L] Ou, W. (2023). Physical Foundations in Equivalent Water Thickness and Crop Water Stress Detection (Doctoral dissertation, The University of Nebraska-Lincoln).

[L] Miao, J., Wang, J., Zhao, D., Shen, Z., Xiang, H., Gao, C., ... & Wu, G. (2024). Modeling strategies and influencing factors in retrieving canopy equivalent water thickness of mangrove forest with Sentinel-2 image. Ecological Indicators, 158, 111497.

[L] Elsherif, A., Gaulton, R., Shenkin, A., Malhi, Y., & Mills, J. (2019). Three dimensional mapping of forest canopy equivalent water thickness using dual-wavelength terrestrial laser scanning. Agricultural and Forest Meteorology, 276, 107627.

[L] Elsherif, A., Gaulton, R., & Mills, J. (2019, July). Measuring leaf equivalent water thickness of short-rotation coppice willow canopy using terrestrial laser scanning. In IGARSS 2019, IEEE International Geoscience and Remote Sensing Symposium (pp. 6087-6090). IEEE.

[L] Martin, R. E., Asner, G. P., Francis, E., Ambrose, A., Baxter, W., Das, A. J., ... & Stephenson, N. L. (2018). Remote measurement of canopy water content in giant sequoias (*Sequoiadendron giganteum*) during drought. Forest Ecology and Management, 419, 279-290.

[L] Xu, X., Konings, A. G., Longo, M., Feldman, A., Xu, L., Saatchi, S., ... & Moorcroft, P. (2021). Leaf surface water, not plant water stress, drives diurnal variation in tropical forest canopy water content. New Phytologist, 231(1), 122-136.

[L] Cheng, T., Riaño, D., & Ustin, S. L. (2014). Detecting diurnal and seasonal variation in canopy water content of nut tree orchards from airborne imaging spectroscopy data using continuous wavelet analysis. Remote Sensing of Environment, 143, 39-53.

[L] Paz‐Kagan, T., & Asner, G. P. (2017). Drivers of woody canopy water content responses to drought in a Mediterranean‐type ecosystem. Ecological Applications, 27(7), 2220-2233.

[L] Marino, E., Yebra, M., Guillén-Climent, M., Algeet, N., Tomé, J. L., Madrigal, J., ... & Hernando, C. (2020). Investigating live fuel moisture content estimation in fire-prone shrubland from remote sensing using empirical modelling and RTM simulations. Remote Sensing, 12(14), 2251.

[L] Adab, H., Devi Kanniah, K., & Beringer, J. (2016). Estimating and up-scaling fuel moisture and leaf dry matter content of a temperate humid forest using multi resolution remote sensing data. Remote Sensing, 8(11), 961.

[L] Quan, X., He, B., Yebra, M., Yin, C., Liao, Z., & Li, X. (2017). Retrieval of forest fuel moisture content using a coupled radiative transfer model. Environmental Modelling & Software, 95, 290-302.

[L] Li, F., Li, Y., Zhang, C., Cheng, Y., Li, Y., & He, L. (2020, September). A Fuel Moisture Content Monitoring Methodology Based on Optical Remote Sensing. In IGARSS 2020, IEEE International Geoscience and Remote Sensing Symposium (pp. 4634-4637). IEEE.

[L] Wang, L., Quan, X., He, B., Yebra, M., Xing, M., & Liu, X. (2019). Assessment of the dual polarimetric sentinel-1A data for forest fuel moisture content estimation. Remote Sensing, 11(13), 1568.

[L] Lai, G., Quan, X., Yebra, M., & He, B. (2022). Model-driven estimation of closed and open shrublands live fuel moisture content. GIScience & Remote Sensing, 59(1), 1837-1856.

[L] Quan, X., He, B., Li, X., & Tang, Z. (2015). Estimation of grassland live fuel moisture content from ratio of canopy water content and foliage dry biomass. IEEE Geoscience and Remote Sensing Letters, 12(9), 1903-1907.

[L] Johnston, K. M. (2024). Evaluation of Linear Fuel Break Systems and Using Remote Sensing Data to Estimate Live Fuel Moisture Content in South-Central Idaho (Doctoral dissertation, University of Idaho).

[L] Tanase, M. A., Nova, J. P. G., Marino, E., Aponte, C., Tomé, J. L., Yáñez, L., ... & Hernando, C. (2022). Characterizing Live Fuel Moisture Content from Active and Passive Sensors in a Mediterranean Environment. Forests, 13(11), 1846.

[L] Costa-Saura, J. M., Balaguer-Beser, Á., Ruiz, L. A., Pardo-Pascual, J. E., & Soriano-Sancho, J. L. (2021). Empirical models for spatio-temporal live fuel moisture content estimation in mixed mediterranean vegetation areas using sentinel-2 indices and meteorological data. Remote Sensing, 13(18), 3726.

[L] Meyer, T., Jagdhuber, T., Piles, M., Fink, A., Grant, J., Vereecken, H., & Jonard, F. (2019). Estimating gravimetric water content of a winter wheat field from L-band vegetation optical depth. Remote Sensing, 11(20), 2353.

*Water potential*

[G] Momen, M., Wood, J. D., Novick, K. A., Pangle, R., Pockman, W. T., McDowell, N. G., & Konings, A. G. (2017). Interacting effects of leaf water potential and biomass on vegetation optical depth. Journal of Geophysical Research: Biogeosciences, 122(11), 3031-3046.

[R] Zhang, Y., Zhou, S., Gentine, P., & Xiao, X. (2019). Can vegetation optical depth reflect changes in leaf water potential during soil moisture dry-down events?. Remote Sensing of Environment, 234, 111451.

[R] Ramoelo, A., Dzikiti, S., Van Deventer, H., Maherry, A., Cho, M. A., & Gush, M. (2015). Potential to monitor plant stress using remote sensing tools. Journal of Arid Environments, 113, 134-144.

[R] Pagay, V., & Kidman, C. M. (2019). Evaluating remotely-sensed grapevine (Vitis vinifera L.) water stress responses across a viticultural region. Agronomy, 9(11), 682.

[L] Holtzman, N. M., Anderegg, L. D., Kraatz, S., Mavrovic, A., Sonnentag, O., Pappas, C., ... & Konings, A. G. (2021). L-band vegetation optical depth as an indicator of plant water potential in a temperate deciduous forest stand. Biogeosciences, 18(2), 739-753.

[L] Jagdhuber, T., Fluhrer, A., Schmidt, A. S., Jonard, F., Chaparro, D., Meyer, T., ... & Entekhabi, D. (2021, July). Retrieval of Forest Water Potential from L-Band Vegetation Optical Depth. In IGARSS 2021, IEEE International Geoscience and Remote Sensing Symposium (pp. 5949-5952). IEEE.

[L] Maciel, D. A., Silva, V. A., Alves, H. M. R., Volpato, M. M. L., Barbosa, J. P. R. A. D., Souza, V. C. O. D., ... & Oliveira dos Santos, J. (2020). Leaf water potential of coffee estimated by landsat-8 images. Plos One, 15(3), e0230013.

[L] Junttila, S., Hölttä, T., Puttonen, E., Katoh, M., Vastaranta, M., Kaartinen, H., ... & Hyyppä, H. (2021). Terrestrial laser scanning intensity captures diurnal variation in leaf water potential. Remote Sensing of Environment, 255, 112274.

[L] Sadeh, R., Avneri, A., Tubul, Y., Lati, R. N., Bonfil, D. J., Peleg, Z., & Herrmann, I. (2024). Chickpea leaf water potential estimation from ground and VENµS satellite. Precision Agriculture, 25(3), 1658-1683.

[L] Beeri, O., May-tal, S., Raz, Y., Rud, R., & Pelta, R. (2018, June). Detecting variability in plant water potential with multi-spectral satellite imagery. In Proceedings of the 14th International Conference on Precision Agriculture, Montreal, Canada (pp. 24-27).

[L] Gao, R., Alsina, M. M., Torres-Rua, A. F., Hipps, L., Kustas, W. P., White, W. A., ... & Gowing, I. (2022, June). Exploratory analysis of vineyard leaf water potential against sUAS multispectral and temperature information. In Autonomous Air and Ground Sensing Systems for Agricultural Optimization and Phenotyping VII (Vol. 12114, pp. 160-185). SPIE.

[L] Delval, L., Bates, J., Jonard, F., & Javaux, M. (2024). Field heterogeneity of soil texture controls leaf water potential spatial distribution in non-irrigated vineyards. EGUsphere, 2024, 1-43.

[L] Bellvert, J., Zarco‐Tejada, P. J., Marsal, J., Girona, J., González‐Dugo, V., & Fereres, E. (2016). Vineyard irrigation scheduling based on airborne thermal imagery and water potential thresholds. Australian Journal of Grape and Wine Research, 22(2), 307-315.

[L] Beeri, O., Pelta, R., Shilo, T., Raz, J., & May-tal, S. (2019). Assessing infield temporal and spatial variability of leaf water potential using satellite imagery and meteorological data. In Precision agriculture’19 (pp. 6647-6666). Wageningen Academic Publishers.

[L] Marusig, D., Petruzzellis, F., Tomasella, M., Napolitano, R., Altobelli, A., & Nardini, A. (2020). Correlation of field-measured and remotely sensed plant water status as a tool to monitor the risk of drought-induced forest decline. Forests, 11(1), 77.

[L] Bellvert, J., Zarco-Tejada, P. J., Girona, J., & Fereres, E. J. P. A. (2014). Mapping crop water stress index in a ‘Pinot-noir’vineyard: comparing ground measurements with thermal remote sensing imagery from an unmanned aerial vehicle. Precision Agriculture, 15, 361-376.

[L] Gao, Y., Zhao, T., Zheng, Z., & Liu, D. (2023). A cotton leaf water potential prediction model based on particle swarm optimisation of the LS-SVM Model. Agronomy, 13(12), 2929.

[L] Easterday, K., Kislik, C., Dawson, T. E., Hogan, S., & Kelly, M. (2019). Remotely sensed water limitation in vegetation: Insights from an experiment with unmanned aerial vehicles (UAVs). Remote Sensing, 11(16), 1853.

[L] Gao, Y., Zhao, T., Zheng, Z., & Liu, D. (2024). Cotton leaf water potential prediction based on UAV visible light images and multi-source data. Irrigation Science, 1-14.

[L] Santos, L. M. D., Ferraz, G. A. E. S., Carvalho, M. A. D. F., Campos, A. A. V., Neto, P. M., Xavier, L. A. G., ... & Rossi, G. (2024). A Spatial Analysis of Coffee Plant Temperature and Its Relationship with Water Potential and Stomatal Conductance Using a Thermal Camera Embedded in a Remotely Piloted Aircraft. Agronomy, 14(10), 2414.

[L] Marques, P., Pádua, L., Sousa, J. J., & Fernandes-Silva, A. (2023). Assessing the water status and leaf pigment content of olive trees: evaluating the potential and feasibility of unmanned aerial vehicle multispectral and thermal data for estimation purposes. Remote Sensing, 15(19), 4777.

[L] Fishman, N., Yungshtein, Y., Yaakobi, A., Obersteiner, S., Rez, L., Mulero, G., ... & Helman, D. (2024). Leaf water potential in a mixed Mediterranean forest from machine learning and unmanned aerial vehicle (UAV)-based hyperspectral imaging. Available at SSRN 4946475.

[L] Wong, C. Y., Gilbert, M. E., Pierce, M. A., Parker, T. A., Palkovic, A., Gepts, P., ... & Buckley, T. N. (2023). Hyperspectral remote sensing for phenotyping the physiological drought response of common and tepary bean. Plant Phenomics, 5, 0021.

[L] Bellvert, J., Marsal, J., Girona, J., & Zarco-Tejada, P. J. (2015). Seasonal evolution of crop water stress index in grapevine varieties determined with high-resolution remote sensing thermal imagery. Irrigation Science, 33, 81-93.

[L] Hassanpour, R., Majnooni-Heris, A., Fard, A. F., & Tasumi, M. (2024). Spatio-temporal monitoring of plant water status using optical remote sensing data and in situ measurements. Advances in Space Research, 74(10), 4688-4704.

[L] Reyes-Rosas, A., Lara-Viveros, F. M., Chávez-Cerón, L., & Khamkure, S. (2023). Estimation of water potential in corn plants using machine learning techniques with UAV imagery and evaluating the effect of flying height. Engineering Proceedings, 56(1), 157.

[L] Tang, Z., Jin, Y., Alsina, M. M., McElrone, A. J., Bambach, N., & Kustas, W. P. (2022). Vine water status mapping with multispectral UAV imagery and machine learning. Irrigation Science, 40(4), 715-730.

[L] Bhandari, S., Raheja, A., Chaichi, M. R., Green, R. L., Do, D., Pham, F. H., ... & Espinas, A. (2018, June). Lessons learned from UAV-based remote sensing for precision agriculture. In 2018 International Conference on Unmanned Aircraft Systems (ICUAS) (pp. 458-467). IEEE.

[L] Cohen, Y., Alchanatis, V., Saranga, Y., Rosenberg, O., Sela, E., & Bosak, A. J. P. A. (2017). Mapping water status based on aerial thermal imagery: comparison of methodologies for upscaling from a single leaf to commercial fields. Precision Agriculture, 18, 801-822.

[L] Rud, R., Cohen, Y., Alchanatis, V., Levi, A., Brikman, R., Shenderey, C., ... & Nigon, T. (2014). Crop water stress index derived from multi-year ground and aerial thermal images as an indicator of potato water status. Precision Agriculture, 15, 273-289.

[L] Gu, S., Liao, Q., Gao, S., Kang, S., Du, T., & Ding, R. (2021). Crop water stress index as a proxy of phenotyping maize performance under combined water and salt stress. Remote Sensing, 13(22), 4710.

[L] Lacerda, L. N. (2021). Using Remote Sensing to Develop Irrigation Scheduling Tools for Variable Rate Irrigation. Dissertation (University of Georgia).

[L] Santos, S. A. D., Ferraz, G. A. E. S., Figueiredo, V. C., Volpato, M. M. L., Machado, M. L., & Silva, V. A. (2022). Evaluation of the water conditions in coffee plantations using RPA. AgriEngineering, 5(1), 65-84.

[L] Santesteban, L. G., Di Gennaro, S. F., Herrero-Langreo, A., Miranda, C., Royo, J. B., & Matese, A. (2017). High-resolution UAV-based thermal imaging to estimate the instantaneous and seasonal variability of plant water status within a vineyard. Agricultural Water Management, 183, 49-59.
